# Solar H_2_ evolution in water with modified diketopyrrolopyrrole dyes immobilised on molecular Co and Ni catalyst–TiO_2_ hybrids†
†Electronic supplementary information (ESI) available: Experimental details, synthetic procedures, additional tables and figures. See DOI: 10.1039/c6sc05219c



**DOI:** 10.1039/c6sc05219c

**Published:** 2017-02-03

**Authors:** Julien Warnan, Janina Willkomm, Jamues N. Ng, Robert Godin, Sebastian Prantl, James R. Durrant, Erwin Reisner

**Affiliations:** a Christian Doppler Laboratory for Sustainable SynGas Chemistry , Department of Chemistry , University of Cambridge , Lensfield Road , Cambridge , CB2 1EW , UK . Email: reisner@ch.cam.ac.uk ; http://www-reisner.ch.cam.ac.uk; b Department of Chemistry , Imperial College London , Exhibition Road , London , SW7 2AZ , UK

## Abstract

A series of diketopyrrolopyrrole (DPP) dyes with a terminal phosphonic acid group for attachment to metal oxide surfaces were synthesised and the effect of side chain modification on their properties investigated. The organic photosensitisers feature strong visible light absorption (*λ* = 400 to 575 nm) and electrochemical and fluorescence studies revealed that the excited state of all dyes provides sufficient driving force for electron injection into the TiO_2_ conduction band. The performance of the DPP chromophores attached to TiO_2_ nanoparticles for photocatalytic H_2_ evolution with co-immobilised molecular Co and Ni catalysts was subsequently studied, resulting in solar fuel generation with a dye-sensitised semiconductor nanoparticle system suspended in water without precious metal components. The performance of the DPP dyes in photocatalysis did not only depend on electronic parameters, but also on properties of the side chain such as polarity, steric hinderance and hydrophobicity as well as the specific experimental conditions and the nature of the sacrificial electron donor. In an aqueous pH 4.5 ascorbic acid solution with a phosphonated DuBois-type Ni catalyst, a DPP-based turnover number (TON_DPP_) of up to 205 was obtained during UV-free simulated solar light irradiation (100 mW cm^–2^, AM 1.5G, *λ* > 420 nm) after 1 day. DPP-sensitised TiO_2_ nanoparticles were also successfully used in combination with a hydrogenase or platinum instead of the synthetic H_2_ evolution catalysts and the platinum-based system achieved a TON_DPP_ of up to 2660, which significantly outperforms an analogous system using a phosphonated Ru tris(bipyridine) dye (TON_Ru_ = 431). Finally, transient absorption spectroscopy was performed to study interfacial recombination and dye regeneration kinetics revealing that the different performances of the DPP dyes are most likely dictated by the different regeneration efficiencies of the oxidised chromophores.

## Abstract 2

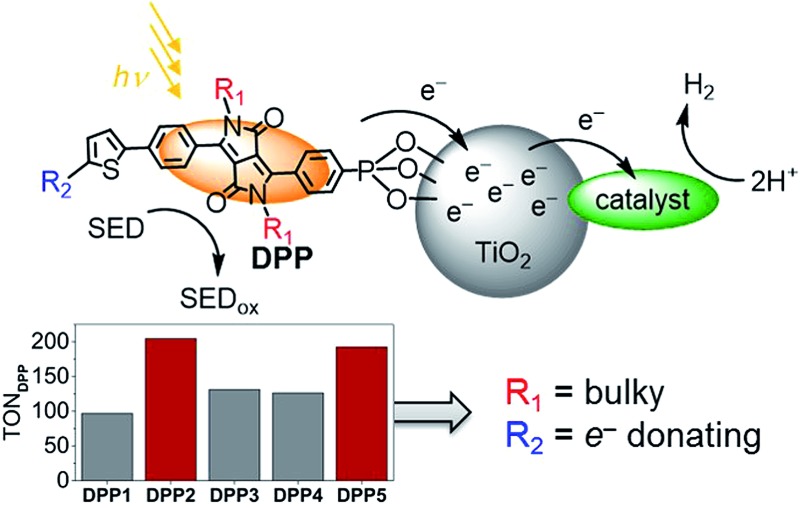
A series of diketopyrrolopyrrole (DPP) dyes with a terminal phosphonic acid group for attachment to metal oxide surfaces were synthesised and the effect of side chain modification on their properties investigated.

## Introduction

**Fig. 1:**
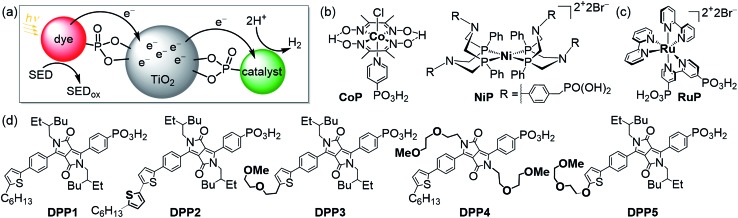
(a) Schematic representation of dye-sensitised photocatalysis (DSP) with a dye and H_2_ evolution catalyst co-immobilised onto TiO_2_ nanoparticles *via* a phosphonate anchoring group (*i.e.*, dye|TiO_2_|catalyst assemblies).^4^ (b) Chemical structures of the molecular H_2_ evolution catalysts **NiP** and **CoP** (a hydrogenase and Pt were also employed as catalysts; see text),^20,21^ (c) the dye **RuP**,^22^ and (d) DPP dyes developed in this study (see Scheme 1 for synthetic route).

Utilising solar energy to split water for the production of renewable hydrogen (H_2_) is a promising strategy to satisfy our demand for sustainable and storable energy.^1–3^ Dye-sensitised photocatalysis (DSP) has emerged as a functional bio-inspired approach for sunlight-driven H_2_ evolution in water by means of co-immobilising a dye and a catalyst on a semiconductor in suspension (Fig. 1a–c),^4^ and this approach can also be adopted in dye-sensitised photoelectrosynthesis cells.^4–13^ DSP systems can be readily assembled through simultaneous attachment of an anchor-bearing molecular photosensitiser and H_2_ evolution catalyst to the surface of an inorganic wide-band gap semiconductor such as TiO_2_.^4,6^ The semiconductor displays dual functionality as it acts as a scaffold for co-immobilisation of the dye and catalyst and, importantly, enables efficient charge separation and accumulation of multiple long-lived, low-potential electrons for catalytic fuel generation.^4,14^ Thus, DSP systems can be regarded as a self-assembled triadic architecture that demonstrates a greater functionality than previously reported homogeneous molecular structures with the added benefit of straightforward assembly from readily available molecular and semiconductor components.^15–19^

To date, DSP has often employed precious metal-containing dyes such as the phosphonated ruthenium tris(bipyridine)-based dye **RuP** (Fig. 1c) anchored to a semiconductor.^20–23^ Despite having beneficial features such as a broad absorption band, a metal-to-ligand charge transfer transition and long-lived charge separated state, ruthenium-dyes challenge future scale-up and low cost applications due to their scarcity, modest molar absorption and the relative lack of simple fine-tuning. Such limitations were also experienced in the past with dye-sensitised solar cell technology, where ruthenium dyes provided benchmark performances for more than a decade.^24^ Recently, organic chromophores (π-conjugated systems) have reached photovoltaic efficiencies of approximately 13% and thereby surpassed Ru-containing photosensitisers.^25^ This is notably due to several advantages of the metal-free chromophores in terms of tunability and strong π–π* transitions. These dyes have been carefully optimised in terms of electronic properties, side chains and engineering of anchoring groups to control the charge transfer processes at the interface with the semiconductor and the redox mediator in an organic electrolyte solution.^26^ Organic dyes are promising candidates for H_2_ evolution *via* DSP if they can demonstrate efficient operation in aqueous solution. Light-driven H_2_ evolution with organic dyes in combination with a metal oxide semiconductor has been previously reported, but these systems required either a Pt co-catalyst, a p-type semiconductor electrode, organic solvents or an anchor-free diffusional dye.^8,27–30^ Only few studies are available with organic chromophores under DSP conditions and even less with commonly used aqueous electron donors, such as triethanolamine (TEOA) or ascorbic acid (AA), or with a molecular catalyst in a semi-heterogeneous photocatalytic scheme.^11,31–34^

Herein, we report the preparation of five phosphonic acid-containing diketopyrrolopyrrole (DPP) photosensitisers bearing different side-chains and electronically active substituents (Fig. 1d). DPP was chosen as chromophore because of its numerous advantages such as well-established synthesis, adjustable photophysical properties and high performances as already reported in optoelectronic devices such as organic transistors and organic and hybrid solar cells.^35–37^

Rational modification of the chromophore architecture provides decisive information for the future preparation of new organic dyes capable of competing with and surpassing the efficiency of Ru dye-based systems. Phosphonic acid was chosen as the anchoring functionality because of its strong attachment to metal oxide surfaces under acidic and pH neutral conditions, whilst allowing electron injection from the excited state of the photosensitiser into the conduction band of TiO_2_.^38^ To the best of our knowledge, these are the first examples of phosphonic acid bearing DPP chromophores, highlighting the chemical compatibility of these two key chemical moieties. The DPP dyes were evaluated using a variety of techniques such as UV-Vis and fluorescence spectroscopy as well as electrochemistry. The photocatalytic activity of the DPP chromophores on TiO_2_ nanoparticles was studied in the presence of the molecular complexes **CoP** or **NiP** (Fig. 1b) as well as a hydrogenase and Pt as H_2_ evolution catalysts in an aqueous sacrificial reaction medium under UV-filtered simulated solar light irradiation.^20,21^ Finally, charge separation and dye regeneration kinetics were investigated by transient absorption spectroscopy. The dye **RuP** (Fig. 1c) was also evaluated under the same conditions to assess the performance of the DPP dyes in comparison to previously established DSP systems.^4^

## Results and discussion

### Synthesis of DPP dyes

**Scheme 1:**
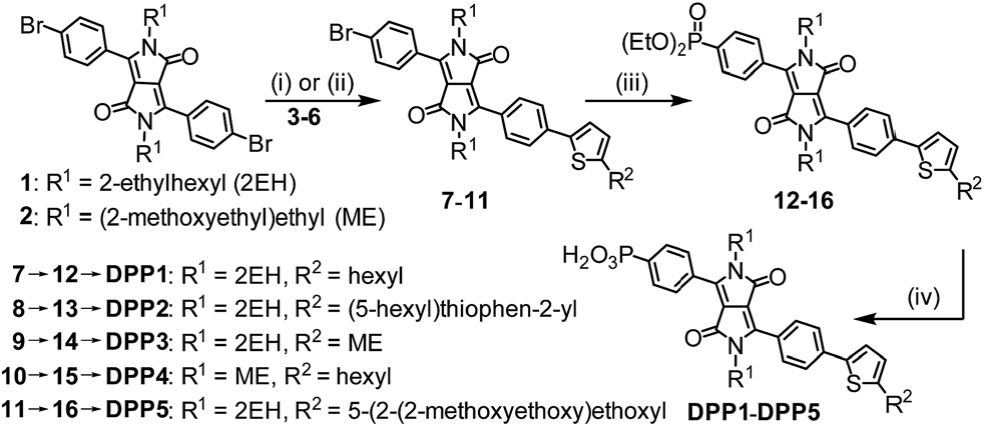
Synthetic route to DPP dyes: (i) [Pd(PPh_3_)_4_], Na_2_CO_3_, THF/H_2_O, 16 h, 70 °C; (ii) [Pd(PPh_3_)_4_], toluene, 16 h, 80 °C; (iii) HPO(OEt)_2_, [Pd(PPh_3_)_4_], Et_3_N, THF, microwave, 120 °C, 0.5 h; (iv) (a) bromotrimethylsilane, DCM, 12 h, r.t. and (b) MeOH/DCM, 2 h, r.t. See ESI† for experimental details and chemical structures of compounds **3–6**.

The synthesis of the DPP dyes is summarised in Scheme 1. The diphenyl DPP core was prepared following a previously described method and consists of a pseudo-Stobbe condensation of 1-bromo-4-cyanobenzene with diethyl succinate.^39^ The *N*-alkylation of the lactams was subsequently achieved in the presence of potassium carbonate and the branched alkyl 1-bromo-2-ethylhexane (Br-2EH) or the linear alkyl 1-bromo-2-(2-methoxyethoxy)ethane (Br-ME), affording compounds **1** or **2**, respectively.^40,41^ These side chains were chosen to increase the general solubility of the dye in organic solvents. Yet, they differ in nature and polarity with the 2EH chain being expected to provide more hydrophobicity and bulkiness to the chromophore. Compounds **1** and **2** were desymmetrised to give the thiophenyl compounds **7** to **11** upon Suzuki–Miyaura or Stille cross-coupling reactions with 1 equiv. of the thiophene derivatives **3** to **6** (see ESI† for chemical structures) in the presence of [Pd(PPh_3_)_4_]. The thiophenyl derivatives **7** to **11** were isolated in moderate yield after purification (28–38%) due to a statistical distribution in cross-coupling products (formation of mono- and bis-coupled adducts).

The phosphonic acid anchoring group was added in two steps. A micro-wave assisted Hirao cross-coupling reaction was performed in the presence of diethyl phosphite to give compounds **12** to **16**, followed by hydrolysis of the corresponding phosphonic esters using bromotrimethylsilane and methanol (MeOH) to give **DPP1** to **DPP5** in good yields. All dyes were soluble in common organic solvents such as MeOH, dichloromethane and tetrahydrofuran and sufficiently soluble in aqueous buffer solutions to allow for their immobilisation on the metal oxide surfaces. The detailed synthetic procedures and characterisation of all compounds (high resolution mass spectrometry, elemental analysis, FT-IR, ^1^H, ^13^C & ^31^P NMR spectroscopy) are available in the ESI.†

### Electronic absorption spectroscopy

**Fig. 2:**
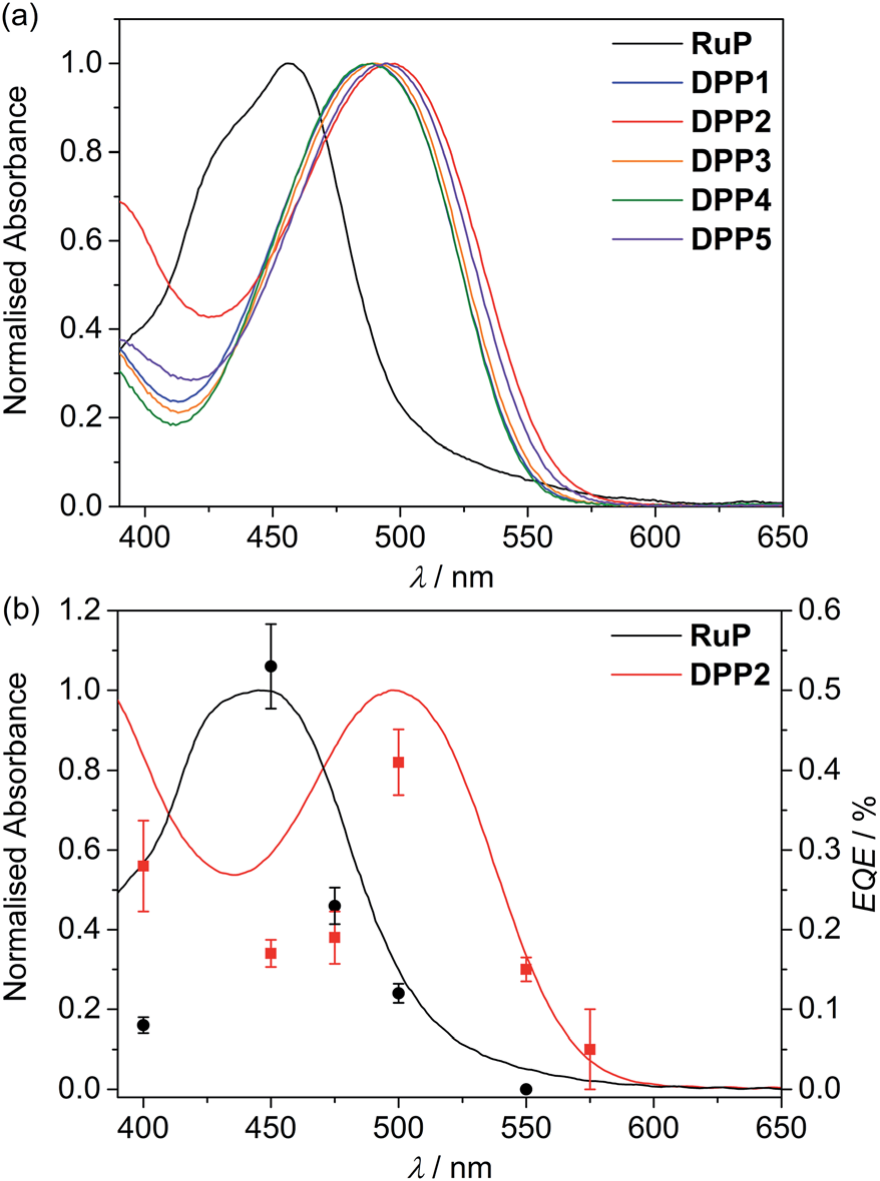
UV-Vis absorption spectra of (a) DPP and **RuP** in DMF solution (see Fig. S1†) and (b) **DPP2** (red trace) and **RuP** (black trace) adsorbed on a thin mesoporous TiO_2_ film at room temperature. The wavelength-dependent EQE values obtained for **RuP**|TiO_2_|**NiP** (black circles) and **DPP2**|TiO_2_|**NiP** (red squares) are also shown. EQE conditions: 2.5 mg TiO_2_, 0.025 μmol of **NiP**, 0.05 μmol of **DPP2** or **RuP** in aqueous AA solution (3 mL, 0.1 M, pH 4.5), 25 °C, 3.03 or 3.15 mW cm^–2^ (see text).

To assess their electronic properties and the impact of the chemical modifications, electronic absorption spectra of the novel DPP dyes were recorded in solution and after chemisorption on transparent mesoporous TiO_2_ films on glass slides (Table 1, Fig. 2 and S1–S3†). *N*,*N*-Dimethylformamide (DMF) was first used as a strong solubilising solvent for solution spectra. In this polar and aprotic solvent, all diphenyl-based DPP chromophores display a strong characteristic absorption centred around 490 nm (*ε*_DPP_ > 1.5 × 10^4^ M^–1^ cm^–1^ at *λ* = 490 nm, Fig. 2a and S1†), matching the solar spectrum maximum intensity wavelength, with all dyes absorbing strongly between 400 and 550 nm.

**Table 1:** Summary of electronic properties and Gibbs energies of the different DPP derivatives and **RuP**

Dye	*λ*_max_ (*ε*)/nm (M^–1^ cm^–1^)	*E*_00_ ^*a*^/eV	*E*(S^+^/S)^*b*^/V *vs.* NHE	*E*(S^+^/S*)^*b*^ ^,^ ^*c*^/V *vs.* NHE	Δ*G*_inj_ ^*d*^/eV		Δ*G*_reg_ ^*e*^/eV	
					pH 4.5	pH 7.0	AA	TEOA
**DPP1**	489 (2.0 × 10^4^)	2.32	1.15	–1.17	–0.62	–0.47	–0.95	–0.33
**DPP2**	496 (2.6 × 10^4^)	2.27	1.10	–1.17	–0.62	–0.47	–0.90	–0.28
**DPP3**	490 (2.3 × 10^4^)	2.32	1.19	–1.13	–0.58	–0.43	–0.99	–0.37
**DPP4**	489 (1.7 × 10^4^)	2.33	1.17	–1.16	–0.61	–0.46	–0.97	–0.35
**DPP5**	494 (1.7 × 10^4^)	2.30	1.01	–1.29	–0.74	–0.59	–0.81	–0.19
**RuP**	457 (1.1 × 10^4^)	1.90 (ref. 42)	1.37	–0.78 (ref. 42)	–0.23	–0.08	–1.17	–0.55

^*a*^ *E*_00_ = (1240/*λ*_abs–fluo_) with *λ*_abs–fluo_ available in ESI (Table S1†).

^*b*^S = ground state of the sensitiser, S* = excited state of the sensitiser, S^+^ = oxidised sensitiser.

^*c*^ *E*(S^+^/S*) = *E*(S^+^/S) – *E*_00_.

^*d*^Δ*G*_inj_ calculated from the equation: Δ*G*_inj_ = *E*(S^+^/S*) – *E*_CB_(TiO_2_) with *E*_CB_(TiO_2_) = –0.70 V *vs.* NHE at pH = 7 and *E*_CB_(TiO_2_) = –0.55 eV *vs.* NHE at pH = 4.5.^43,44^

^*e*^Δ*G*_reg_ calculated from the equation: Δ*G*_reg_ = –(*E*(S^+^/S) – *E*(SED^+^/SED)) with *E*(SED^+^/SED)_AA_ = 0.20 V *vs.* NHE^45^ and *E*(SED^+^/SED)_TEOA_ = 0.82 V *vs.* NHE.^46^

In a polar protic solvent such as MeOH, no significant spectral differences were observed between **DPP1**, **DPP3** and **DPP4** as their structures only differ in electronically inactive side-chains (Fig. S2a†). However, slight bathochromic shifts were observed for the **DPP5** and **DPP2** absorption maxima as a result of the strong electron-donating *O*-substituted side-chain and increased conjugation length from the second thiophene unit, respectively. More substantial spectral differences were observed between the different DPP dyes in non-polar aprotic solvents such as toluene (Fig. S2b†). Due to enhanced molecular solubility, **DPP2** features a sharper absorption peak in toluene than in MeOH, whereas the more hydrophilic **DPP4** exhibits a broader absorption most likely due to dye aggregation/organisation. Such behaviour indicates that the side-chains' nature/polarity directly affects its interaction with the media, thereby potentially modulating electronic communication with the electrolyte components (*e.g.* proton source or electron donating reagent).

Immobilisation of the DPP photosensitisers on TiO_2_ films allowed for better insight into the absorption ability of the light-harvesting system in a state closer to the photocatalytic DSP conditions. As a result of a slight dye aggregation, absorption peaks are marginally broadened towards higher wavelengths, reaching 575 nm in the case of **DPP2** (Fig. S3†). Among all dyes, **DPP2** displays the broadest light-harvesting window, which potentially allows more photons to be collected, and consequently gives rise to a higher electron injection probability. As a comparison, the phosphonated Ru-dye **RuP** exhibits strong absorption close to the UV region with a sharp onset around 515 nm (Fig. 2a and S3†). The maximum intensity of the metal-to-ligand charge transfer (MLCT) transition in **RuP** was recorded at 457 nm, with a weaker molar absorption intensity (*ε* **_RuP_** ≈ 1.1 × 10^4^ M^–1^ cm^–1^) compared to the DPP dyes.

### Emission spectroscopy

In order to gain information about the dyes' singlet excited state (S_1_), the steady-state photoluminescence of the DPP dyes was recorded in DMF solution at room temperature. The zero–zero excitation energies (*E*_00_) were estimated at the intersection between the normalised absorption and luminescence spectra^40^ (Fig. S4†) and the results are summarised in Tables 1 and S1.†

Photoexcitation at 460 nm results in a strong emission for all dyes with a maximum centred at approximately 570 nm (±10 nm). In the case of **DPP1**, **DPP3** and **DPP4**, similar *E*_00_ values were obtained (*E*_00_ ≈ 2.32 eV). Slightly smaller values were determined for **DPP2** and **DPP5** (*E*_00_ ≈ 2.30 eV), in line with their red-shifted absorption characteristics. Medium Stokes shifts were recorded for all dyes (Δ*ν̄* = 2500 to 3000 cm^–1^), indicating notable reorganisation of the dyes' dipole moment in the excited state. The reorganisation could originate from the diminution of the thiophene-phenyl angle as previously reported.^40^ Among all the dyes, **DPP2** and **DPP5** featured the largest shifts, which could also suggest a stronger ability for efficient charge transfer as the result of a major change between the excited and ground state dipoles.

### Electrochemical properties

The redox potentials of the DPP dyes were recorded by cyclic voltammetry after immobilisation on mesoporous indium tin oxide electrodes^47^ in an acetonitrile solution containing tetrabutylammonium tetrafluoroborate (0.1 M) as electrolyte (Table 1). Cyclic voltammetry shows that all DPP dyes exhibit an irreversible oxidation wave, located at approximately *E*_onset_ ≈ 1.17 V *vs.* NHE (onset potential)^48^ for **DPP1**, **DPP3** and **DPP4** (Fig. S5†). The marginal differences between the oxidation potential of these three photosensitisers confirm the minor impact of side chain modification on the electronic properties with the main electrochemical processes being localised on the DPP core. The onset of the oxidation wave is shifted to *E*_onset_ = 1.01 V *vs.* NHE in the case of **DPP5**, where the electron donating, *O*-substituted thiophene facilitates the chromophore oxidation. **DPP2** also exhibits a less positive oxidation potential at *E*_onset_ = 1.10 V *vs.* NHE, most likely due to the additional thiophene unit. This difference could have a significant influence on the regeneration ability of an oxidised dye by a chemical reductant such as a sacrificial electron donor (SED) in photocatalytic schemes (see transient absorption spectroscopy study below).

TEOA (pH 7) and AA (pH 4.5) have been used as SEDs in this study. The driving force for dye regeneration (Δ*G*_reg_) following oxidative quenching of each dye's excited state by TiO_2_ was estimated. The low redox potential of AA (*E*(SED^+^/SED)_AA_ < 0.20 V *vs.* NHE, pH 4.5)^45,49–51^ allows it to thermodynamically act as a strong electron-donating reagent for all oxidised dyes, generating a highly favourable regeneration reaction (Δ*G*_reg_ < –0.80 eV). However, TEOA's more positive redox potential for oxidation (*E*(SED^+^/SED)_TEOA_ = 0.82 V *vs.* NHE, pH 7)^46^ provides considerably less driving force, implying a potentially sluggish regeneration of DPP dyes (Δ*G*_reg_ ≈ –0.19 to –0.37 eV). This driving force differs from **RuP** (*E*_onset_ = 1.37 V *vs.* NHE, Table 1), where a sufficiently exergonic situation (Δ*G*_reg_ ≈ –0.55 eV) is expected to allow an efficient reaction with TEOA.

The addition of *E*_00_ to the dye's *E*(S^+^/S) provides an estimate for the excited state oxidation potential *E*(S^+^/S*) of the DPP dyes relevant for oxidative quenching. Apart from **DPP5**, comparable values were obtained for all DPP dyes (*E*(S^+^/S*) ≈ –1.15 V *vs.* NHE), indicating that the different substituents have little influence on the excited state energy levels. In the case of the alkyloxy-functionalised **DPP5**, a more negative *E*(S^+^/S*) of approximately –1.29 V *vs.* NHE was obtained, which shows that the electron donating group affected both the HOMO and LUMO energy levels. The driving force for electron injection, Δ*G*_inj_, from the dye's excited state to the TiO_2_ conduction band (CB; *E*_CB_(TiO_2_) = (–0.29 V – 0.059 V × pH) *vs.* NHE)^43,44^ was calculated for both pH values and proved to be sufficient for all DPP photosensitisers (Δ*G*_inj_ < –0.4 eV).

### Photocatalysis of DPP|TiO_2_ with molecular catalysts

The DPP dyes were co-immobilised with the molecular H_2_ evolution catalysts **CoP** or **NiP** (Fig. 1b) on TiO_2_ *via* their phosphonic acid functionalities.^4,20,21,52^ The DSP systems were self-assembled by dispersing Evonik P25 TiO_2_ nanoparticles in a buffered and SED-containing aqueous solution, followed by addition of the molecular catalyst and dye to give the dye|TiO_2_|catalyst assemblies (see ESI† for details). In a standard experiment, 2.5 mg of TiO_2_ and 0.05 μmol of dye were used in 3 mL of aqueous SED solution with different amounts of either **CoP** or **NiP** in a sealed photoreactor (headspace = 4.84 mL). The photocatalytic activity of the DPP|TiO_2_|catalyst assemblies was studied in the presence of the SED TEOA (pH 7.0 with **CoP**) or AA (pH 4.5 with **NiP**) under previously identified optimal conditions for the applied molecular catalysts.^20,21^ The deaerated DPP|TiO_2_|**CoP** and DPP|TiO_2_|**NiP** suspensions were kept at 25 °C and irradiated with UV-filtered simulated solar light (AM 1.5G, 100 mW cm^–2^, *λ* > 420 nm). The UV cut-off filter prevented band gap excitation of TiO_2_ and ensured that light is only harvested by the molecular dye. The corresponding **RuP**-based system, **RuP**|TiO_2_|catalyst, was also studied for comparison.

Binding of **CoP**, **NiP** and **RuP** to TiO_2_ has been studied previously and a maximum loading capacity of approximately 0.15 μmol of molecular components on 2.5 mg of TiO_2_ was determined.^20,21^ Attachment of the DPP dyes to TiO_2_ particles under experimental conditions was confirmed for **DPP1** and **DPP4**. 0.05 μmol of dye were stirred with 2.5 mg of TiO_2_ in aqueous AA or TEOA solution. The modified TiO_2_ particles were separated *via* centrifugation and UV-Vis spectroscopy of the supernatant showed that more than 80% of DPP dye was adsorbed onto the particles (Table S2†), ensuring a strong light-harvesting ability for both the most hydrophilic (**DPP4**) and the corresponding hydrophobic (**DPP1**) dye on TiO_2_.

Control experiments established that no H_2_ or only trace amounts were produced in the absence of either DPP dye, TiO_2_, molecular catalyst, light, or SED. No H_2_ was also detected, in presence of ZrO_2_ nanoparticles, instead of TiO_2_ (Table S3†), due to the high-energy level of the CB of ZrO_2_ (*E*_CB_(ZrO_2_) = (–1.0 V – 0.059 V × pH) *vs.* NHE) preventing the electron injection from the excited state of **DPP1** and **DPP4**.^53^ Upon addition of potassium phosphate (0.1 M, pH adjusted to corresponding SED solution) to the aqueous SED solution, no H_2_ was produced by the DSP systems due to displacement of the molecular components from the TiO_2_ surface by excess phosphate ions,^45,54^ demonstrating that a homogeneous reductive quenching pathway is not contributing to H_2_ evolution. Experiments in the presence of the corresponding Co and Ni metal salts instead of the molecular catalysts showed no H_2_ evolution (Table S3†). Thus, all components of the DSP are vital for light-driven H_2_ evolution. It further demonstrates that the system proceeds *via* oxidative quenching of DPP and a ‘through particle’ electron transfer mechanism requiring TiO_2_ as an electron mediator,^21^ and only immobilised molecular components on a semiconductor with suitable band energies (*e.g.*, TiO_2_) can contribute to the photoactivity.

**Fig. 3:**
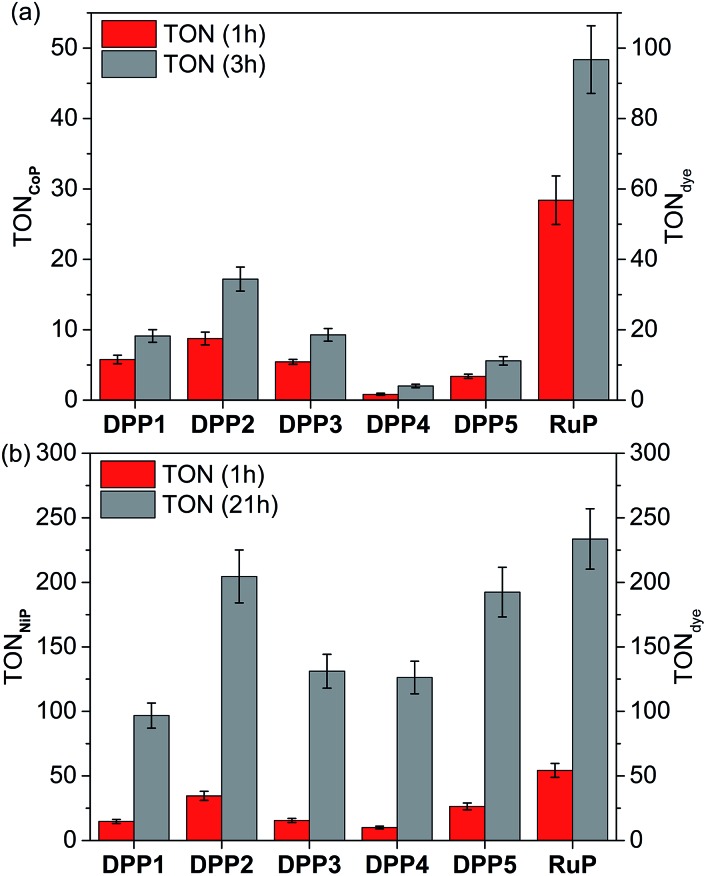
Photocatalytic H_2_ evolution with (a) DPP|TiO_2_|**CoP** and (b) DPP|TiO_2_|**NiP** in comparison with the analogous **RuP** system. Conditions: 2.5 mg TiO_2_, 0.05 μmol dye and 0.05 μmol **CoP** or 0.025 μmol **NiP** in either aqueous TEOA solution (0.1 M, pH 7, **CoP**) or AA solution (0.1 M, pH 4.5, **NiP**) under UV-filtered simulated solar light irradiation (AM 1.5G, 100 mW cm^–2^, *λ* > 420 nm) at 25 °C.

The photocatalytic activity of DPP|TiO_2_|**CoP** was studied in TEOA solution (0.1 M) at pH 7 and the results are summarised in Tables 2, S4 and S5,† and Fig. 3a, S6 and S7.† The TiO_2_ nanoparticles were loaded with **CoP** and the corresponding DPP dye (0.05 μmol each) and the amount of **CoP** optimised for a maximum **CoP**-based turnover number (TON**_CoP_**). Among the DPP|TiO_2_|**CoP** assemblies, a maximum turnover frequency, TOF**_CoP_** of 8.8 ± 0.9 h^–1^ and TOF_DPP_ = 17.5 ± 1.8, was obtained with **DPP2**. Only trace amounts of H_2_ were observed with **DPP4**, whereas a TOF_DPP_ of 6 to 12 h^–1^ was achieved with **DPP1**, **DPP3** and **DPP5**.

**Table 2:** Photocatalytic performance of DSP systems studied in this work^*a*^

System	TOF_cat_ ^*f*^/h^–1^	TOF_dye_ ^*g*^/h^–1^	*n*(H_2_)/μmol (1 h)	TON_cat_ ^*f*^	TON_dye_ ^*g*^
**Dye|TiO**_**2**_ **|** ****CoP**** ^*b*^					
**DPP2**	8.8 ± 0.9	17.5 ± 1.8	0.43 ± 0.04	17.2 ± 1.7 (3 h)	34.4 ± 3.4 (3 h)
**RuP**	28.4 ± 3.4	56.8 ± 6.9	1.42 ± 0.17	48.4 ± 4.8 (3 h)	96.7 ± 10.0 (3 h)

**Dye|TiO**_**2**_ **|** ****NiP**** ^*c*^					
**DPP1**	14.7 ± 1.5	14.7 ± 1.5	0.38 ± 0.04	96.8 ± 9.7 (21 h)	96.8 ± 9.7 (21 h)
**DPP2**	34.6 ± 3.5	34.6 ± 3.5	0.86 ± 0.09	204.6 ± 20.5 (21 h)	204.6 ± 20.5 (21 h)
**DPP3**	15.5 ± 1.6	15.5 ± 1.6	0.39 ± 0.04	131.1 ± 13.1 (21 h)	131.1 ± 13.1 (21 h)
**DPP4**	10.0 ± 1.0	10.0 ± 1.0	0.25 ± 0.03	126.3 ± 12.6 (21 h)	126.3 ± 12.6 (21 h)
**DPP5**	26.4 ± 2.6	26.4 ± 2.6	0.66 ± 0.07	192.4 ± 19.2 (21 h)	192.4 ± 19.2 (21 h)
**RuP**	54.3 ± 5.4	54.3 ± 5.4	1.35 ± 0.14	233.6 ± 23.4 (21 h)	233.6 ± 23.4 (21 h)

**Dye|TiO**_**2**_ **|H**_**2**_ **ase** ^*d*^					
**DPP2**	8650 ± 1100	17.3 ± 2.2	0.43 ± 0.06	87 600 ± 11 100 (21 h)	175 ± 22 (21 h)
**RuP**	12 500 ± 1246	25.0 ± 2.5	0.62 ± 0.06	91 100 ± 22 300 (21 h)	182 ± 45 (21 h)

**Dye|TiO**_**2**_ **|Pt** ^*e*^					
**DPP2**	n.d.^*h*^	337 ± 33.7	8.4 ± 0.8	n.d.^*h*^	2660 ± 265 (24 h)
**RuP**	n.d.^*h*^	71.3 ± 7.1	1.8 ± 0.2	n.d.^*h*^	431 ± 95 (24 h)

^*a*^General conditions: samples contained dye and catalyst loaded onto P25 TiO_2_ nanoparticles (2.5 mg) in a total volume of 3 mL of SED solution and were irradiated with UV-filtered simulated solar light (100 mW cm^–2^, AM 1.5G, *λ* > 420 nm) at 25 °C.

^*b*^ **CoP** (0.05 μmol) and dye (0.05 μmol) on TiO_2_ in aqueous TEOA solution (3 mL, pH 7, 0.1 M), see Table S5 for results for **DPP1**, **DPP3**, **DPP4** and **DPP5**.

^*c*^ **NiP** (0.025 μmol) and dye (0.05 μmol) on TiO_2_ in aqueous AA solution (3 mL, pH 4.5, 0.1 M).

^*d*^[NiFeSe]-H_2_ase (50 pmol) and dye (0.05 μmol) on TiO_2_ in AA-MES solution (3 mL, pH 6, 0.1 M each).

^*e*^Pre-platinised TiO_2_ (2.5 mg) and dye (0.05 μmol) in aqueous AA solution (3 mL, pH 4.5, 0.1 M).

^*f*^TOF_cat_ and TON_cat_ were calculated as follows: TOF_cat_ = *n*(H_2_) after 1 h/*n*(catalyst) and TON_cat_ = *n*(H_2_) after x h/*n*(catalyst).

^*g*^TOF_dye_ and TON_dye_ were calculated as follows: TOF_dye_ = 2*n*(H_2_) after 1 h/*n*(dye) and TON_dye_ = 2*n*(H_2_) after x h/*n*(dye).

^*h*^Not determined due to the unknown amount of catalytically active sites; control experiments and optimisations of DSP systems are listed in Tables S3 to S7.

The results for the DPP dyes are in approximate accordance with trends expected from the electronic properties. The slightly better performance of **DPP2** may be due to its broader light absorption window and the poor performance of **DPP5** due to the smallest Δ*G*_reg_. For **DPP4**, the linear hydrophilic core side chain appears to have a negative impact on the performance of the dye at pH neutral conditions. This effect could be explained by the formation of a dense packed layer of dye that induced a steric effect possibly preventing the dye regeneration from the SED.^31^ This is confirmed to some extent by the higher loading of **DPP4** than **DPP1** (Table S2†). Furthermore, the similar performances observed for **DPP1** and **DPP3** (≈11 h^–1^) indicate a minimal impact of the thiophene's “tailing” side-chain hydrophilicity under the employed conditions.

In general, the lower performance of the DPP-based DSP systems compared to **RuP**|TiO_2_|**CoP** (TOF**_CoP_** = 28.4 ± 3.4 h^–1^, TOF**_RuP_** = 56.8 ± 6.9 h^–1^) can be attributed to the small driving force for regeneration of the oxidised dye (Δ*G*_reg_ > –0.35 eV) by TEOA after electron transfer from the excited dye to the TiO_2_-CB. In agreement, we observe a significant bleaching of the orange colouration of the DPP-sensitised TiO_2_ nanoparticles as a result of dye degradation in the absence of an efficient electron regeneration process after one hour of light exposure. This correlates well with the observed cessation of photo-H_2_ generation of DPP|TiO_2_|**CoP** within the first hours of irradiation (Fig. 3a and S7,† Tables 2 and S5†).

The DPP dyes were subsequently studied with the molecular H_2_ evolution catalyst **NiP** co-adsorbed on TiO_2_ nanoparticles in an aqueous pH 4.5 AA solution (0.1 M). The amount of **NiP** (0.025 μmol) was optimised for a maximum TON**_NiP_** (Table S6 and Fig. S8†). The following trend based on TOF**_NiP_** and TOF_DPP_ was observed for DPP|TiO_2_|**NiP**: **DPP2** > **DPP5** > **DPP3** ≈ **DPP1** > **DPP4** (Fig. 3b and S9†, Tables 2 and S7†), with **DPP2** achieving the highest TOF**_NiP_**_/DPP_ of 34.6 ± 3.5 h^–1^. With **DPP2** and **DPP5**, TON**_NiP_**_/DPP_ of 204.6 ± 20.5 and 192.4 ± 19.2 were obtained after 21 h of visible light irradiation, comparing well with the corresponding **RuP**-based DSP (TON**_NiP_**_/DPP_ of 233.6 ± 23.4). The DPP dyes therefore exhibit a good stability, allowing for prolonged H_2_ generation with high performance in DSP with **NiP**.

The large driving force available for regeneration of DPP^+^ (Δ*G*_reg_ < –0.80 eV) when using AA as SED is likely a key reason for the better performance of the DPP dyes in aqueous AA compared to TEOA solution.

The DSP systems with **DPP1**, **DPP3** and **DPP4** feature a similar TOF_DPP_ (10 to 15 h^–1^), which agrees with their almost identical electronic properties. However, long-term irradiation (21 h) of the **DPP3**- and **DPP4**-based systems results in a higher TON_DPP_ than with **DPP1** (Tables 2 and S7,† Fig. 3b and S9†). This observation suggests that the side chains' polarity has a secondary but not negligible impact on the dye stability/efficiency with a synergistic relationship between the nature of the SED and/or pH variation. While bulky lipophilic chains positioned on the core (the oxidation centre) appear advantageous to the system under pH neutral conditions (TON**_DPP1_** ≈ TON**_DPP3_** > TON**_DPP4_**, see above), the presence of hydrophilic chains appeared to be beneficial (TON**_DPP3_** ≈ TON**_DPP4_** > TON**_DPP1_**) at pH 4.5. The better performance of **DPP4** at pH 4.5 compared to pH 7 is presumably due to the concomitant binding of AA to the TiO_2_ surface, thereby preventing strong deleterious aggregation of the DPP dye (see above).

The additional *O*-donor functionality in **DPP5** presumably accounts for the better performance compared to **DPP3** due to improved charge separation properties as observed in fluorescence and UV-Vis experiments. This is in contrast to the performance at pH 7, where **DPP3** performed better than **DPP5**. Under pH neutral conditions, the small Δ*G*_reg_ is presumably the limiting factor (–0.37 *vs.* –0.19 eV), whereas Δ*G*_reg_ is sufficiently large at pH 4.5 (<–0.8 eV) that other parameters like the push–pull architecture dominate the performance. Similarly, in the case of **DPP2**, the extended absorption window allows for better light absorption resulting in the highest performance amongst all DPP|TiO_2_|**NiP** assemblies.

In contrast to the DPP-based DSP systems studied herein, there are two mechanistic H_2_ evolution pathways possible for **RuP**|TiO_2_|**NiP** in AA (Fig. S10†).^21,45^ In addition to the ‘through particle’ pathway, where RuP* is oxidatively quenched by the semiconductor CB, reductive quenching of RuP* by AA is also possible. In the latter case, a strongly reducing dye species (**RuP^–^**) is formed, which can directly reduce **NiP** to initiate H_2_ evolution through an ‘on particle’ pathway.^21^ This might account for the higher TOF**_NiP_** of **RuP**|TiO_2_|**NiP** as two pathways contribute toward H_2_ production as opposed to a pure ‘through particle’ pathway in DSP with the DPP dyes (see above).

### External quantum efficiency

The external quantum efficiency (EQE) was determined for **DPP2**|TiO_2_|**NiP** at different wavelengths and compared to **RuP**|TiO_2_|**NiP**. The obtained EQE values match well with the absorption profiles of the dyes on TiO_2_ giving the highest value at their corresponding absorption maxima (Fig. 2b and Table S8†). Notably, **RuP**|TiO_2_|**NiP** did not show any photo-H_2_ activity at *λ* = 550 nm, whereas **DPP2**|TiO_2_|**NiP** was still active at this wavelength (EQE ≈ 0.15%). This highlights the good solar light absorption properties of **DPP2** and confirms that light from a wide range of the visible spectrum can successfully be used for H_2_ evolution in **DPP2**|TiO_2_|**NiP**.

An EQE of approximately 0.3 and 0.4% was achieved with **DPP2**|TiO_2_|**NiP** at *λ* = 400 and 500 nm, respectively, which is in the same range as previously reported EQE values of molecular DSP systems with **RuP**.^45,54^ All EQE values were recorded using the standard conditions from the photocatalysis experiments and no optimisation was performed. EQEs represent a lower limit for an internal quantum yield, which would assume that all incident light was absorbed by the sample.

### Photocatalysis of DPP|TiO_2_ with hydrogenase and Pt

We also studied the best performing DPP chromophore, **DPP2**, in combination with previously established benchmark catalysts, which have been applied in DSP systems, *i.e.* the reversible H_2_ cycling catalysts hydrogenase (H_2_ase)^55,56^ and Pt.^22,32^ Using hydrogenase allows establishing the biocompatibility of DPP dyes and Pt as a catalyst eliminates or at least substantially reduces kinetic limitations from catalyst turnover and allows for evaluation of the true potential of the organic dyes.

For experiments with hydrogenase, P25-TiO_2_ (2.5 mg) was loaded with **DPP2** or **RuP** (0.05 μmol) in an aqueous AA-MES solution (3 mL, 0.1 M each, pH 6, MES = 2-(*N*-morpholino)ethanesulfonic acid) and a [NiFeSe]-H_2_ase from *Desulfomicrobium baculatum* (50 pmol) was added to the deaerated suspension.^57^ This hydrogenase was selected for its well-established properties as highly active H_2_ evolution catalyst that displays O_2_-resistance paired with little inhibition by H_2_ and its excellent attachment to metal oxide surfaces.^58,59^ Pt was pre-deposited on P25 TiO_2_ nanoparticles^22^ and the modified particles (2.5 mg) were sensitised with either **DPP2** or **RuP** (0.05 μmol) after suspending the particles in aqueous AA solution (3 mL, 0.1 M, pH 4.5). As for experiments performed with **CoP** and **NiP**, all samples were stirred at 25 °C and irradiated with UV-filtered simulated solar light (100 mW cm^–2^, AM 1.5G, *λ* > 420 nm).

**Fig. 4:**
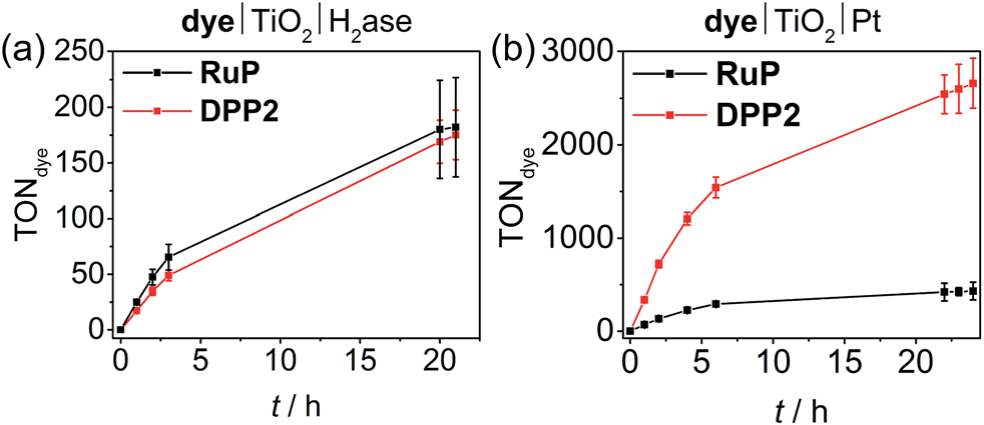
(a) Photocatalytic activity of **DPP2**|TiO_2_|H_2_ase and **RuP**|TiO_2_|H_2_ase. Conditions: 2.5 mg TiO_2_, 50 pmol [NiFeSe]-H_2_ase, 0.05 μmol of **DPP2** or **RuP**, in 3 mL AA-MES solution (0.1 M, pH 6); (b) photocatalytic activity of **DPP2**|TiO_2_|Pt and **RuP**|TiO_2_|Pt. Conditions: 2.5 mg pre-platinised TiO_2_, 0.05 μmol of **DPP2** or **RuP**, in 3 mL AA solution (0.1 M, pH 4.5). In both cases the samples were irradiated with UV-filtered solar light (100 mW cm^–2^, AM 1.5G, *λ* > 420 nm) at 25 °C.

Similar to the **NiP**-based DSP systems, **RuP**|TiO_2_|H_2_ase and **DPP2**|TiO_2_|H_2_ase displayed similar photoactivity (TON**_DPP2_** = 175 ± 22 and TON**_RuP_** = 182 ± 45, Fig. 4a, Tables 2 and S9†). This result may originate from the low amount of H_2_ase available at the TiO_2_ surface, generating a catalysis-limited system. However, the activity of the **DPP2**-based system (TOF_enzyme_ ≈ 8.7 × 10^3^) compares well with a previously reported carbon nitride|TiO_2_|H_2_ase hybrid,^56^ confirming a good compatibility of the DPP chromophore with the biocatalyst.

When using Pt as H_2_ evolution catalyst, the **DPP2**-containing assembly significantly outperforms **RuP**|TiO_2_|Pt, achieving a TOF_dye_ of 337 ± 33.7 and 71.3 ± 7.1, respectively (Fig. 4b, Tables 2 and S10†). Notably, the **DPP2**|TiO_2_|Pt was also found to be considerably more efficient with a TON_dye_ of 2660 ± 265 after 24 h of irradiation, whereas a TON**_RuP_** of only 431 ± 95 was observed for **RuP**. The higher efficiency of the DPP-based system could stem from altered kinetic pathways. With Pt being a fast H_2_ evolution catalyst, the systems are less limited by charge recombination kinetics (see transient absorption spectroscopy), but more likely by the number of available CB electrons in TiO_2_ – this is a direct consequence of the DPP photosensitisers' enhanced light-harvesting and electron injection abilities.

### Transient absorption spectroscopy

We performed transient absorption spectroscopy (TAS) measurements to evaluate both the charge recombination and dye regeneration processes. To reach high efficiencies, the productive charge transfer steps must compete favourably with the undesired energy loss pathways. For example, electron injection should occur faster than excited state relaxation, and oxidised dye regeneration should be faster than charge recombination.^60^

**Fig. 5:**
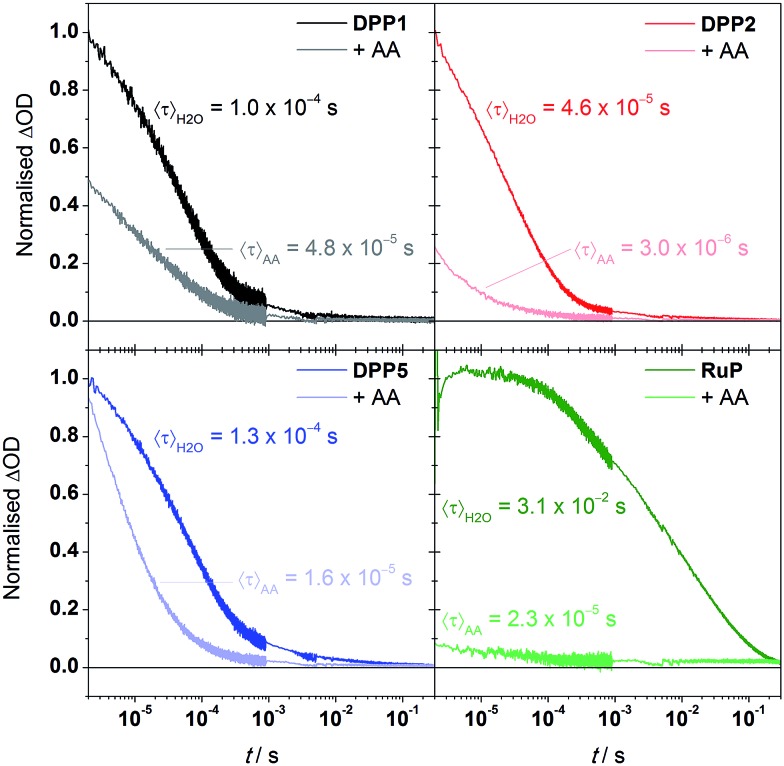
Normalised change in absorbance at 700 nm in H_2_O or AA solutions (10 mM, pH = 4.5) of dye-sensitised TiO_2_ thin films. Traces were normalised to the amplitude observed in H_2_O at 2 μs for the DPP dyes, and 3 μs for **RuP**. Characteristic mean lifetimes are indicated near the corresponding trace.

We monitored the charge-separated state produced upon the photoexcitation at 500 nm of DPP-sensitised TiO_2_ films by following the transient change in absorption at 700 nm, assigned to photogenerated dye cation absorption. Normalised results for **DPP1**, **DPP2**, and **DPP5** are shown in Fig. 5 (see Fig. S11† for non-normalised traces). Measurements were attempted for **DPP3** and **DPP4**, but these dyes proved to be highly unstable under the TAS conditions in the absence of a SED (it is likely that chemical transformations following photo-oxidation of the dyes causes the instability). We expect the extinction coefficients of the oxidised DPP dyes to be similar on the basis of the similar ground state optical properties. We may thus compare the initial signal amplitude, proportional to the concentration of oxidised DPP produced, observed for the different DPP dyes. The initial amplitudes at 2 μs will be related to the charge injection yield and is the highest for **DPP5**, consistent with its larger Δ*G*_inj_ compared to **DPP1** and **DPP2**. A decrease of 20% is seen for **DPP1** compared to **DPP2**. As the two dyes possess the same Δ*G*_inj_, the change potentially reflects differential dye orientation or polarity of the side chains. **DPP1** shows the lowest initial amplitude, which might explain its lower photoactivity compared to **DPP2** and **DPP5**.

The decays presented in Fig. 5 could be well-described by a stretched exponential expression (fits shown in Fig. S11†), in line with the dispersive recombination kinetics observed in TiO_2_ caused by charge trapping/detrapping.^61,62^ We characterised the lifetime of the charge separated state from the mean lifetime 〈*τ*〉 obtained from fitting (see ESI†). All three DPP dyes show decays comparable to previous reports of DPP-sensitised TiO_2_,^63^ and have similar charge-separated lifetimes near 100 μs, suggesting that the observed differences in activity between dyes are not due to changes in this recombination lifetime. The excited state dynamics of the DPP photosensitisers on TiO_2_ were also compared to **RuP** (excitation at 450 nm, monitoring at 700 nm). In line with previous investigations,^21^ the transient signal decays on the millisecond timescale. The mean lifetime for **RuP** was 31 ms, roughly 300-fold longer than observed for the DPP-based dyes. The increased charge separation lifetime is possibly due to decreased electronic coupling or an increased spatial charge separation between the photosensitiser cation and the TiO_2_ surface in the case of **RuP**.^64^

We next performed TAS measurements in the presence of AA (10 mM) to investigate dye regeneration (Fig. 5). Quenching of the oxidised dyes was confirmed by observation of a reduced signal amplitude and shortening of 〈*τ*〉, both for the DPP dyes and **RuP**. The reduction in initial signal amplitude showed large variations between DPP dyes, ranging from less than 10% for **DPP5** to 75% for **DPP2**, suggesting faster and efficient (>90%) regeneration for the latter. The shape of the decays indicates that for **DPP1** and **DPP2** dye regeneration mainly takes place on the sub-μs timescale while the same process takes place in approximately 10 μs for **DPP5**. We calculated the regeneration efficiencies from the competitive kinetics of regeneration and charge recombination (see ESI† for details): the regeneration is most efficient (94% yield) for **DPP2**, which may partly be the reason for its best performance in DSP. Although the regeneration kinetics are significantly slower in the case of **DPP5**, regeneration is relatively efficient (87% yield) as competition with charge recombination is still favourable, and is in line with the comparable photoactivity to **DPP2**. **DPP1** showed the lowest regeneration yield, 52%, another factor that may limit its photoactivity. The regeneration yields do not correlate directly with Δ*G*_reg_ and appear to rely primarily on other factors such as the dyes' hydrophobicity, orientation or push–pull architecture.^65,66^

Comparative experiments with **RuP** showed a more significant sub-μs quenching of the oxidised dye, with the initial amplitude decreasing by over 90% in the presence of AA, and overall shows quantitative regeneration. The more efficient regeneration with **RuP** is consistent with its larger Δ*G*_reg_, and consistent with the slightly higher photoactivity obtained for this dye in the systems without Pt.

Despite the high regeneration yields, overall quantum efficiencies of the hybrid systems **RuP**|TiO_2_|**NiP** and **DPP2**|TiO_2_|**NiP** are below 1%. This discrepancy can be explained by the increased electron density in the CB of TiO_2_ under continuous irradiation, which will lead to faster charge recombination kinetics that reduces the regeneration yield in bulk photocatalysis experiments.^67^ We have previously determined that the first reduction of molecular catalyst on **RuP**-sensitised TiO_2_ occurs on the μs to ms timescales.^14,21,54^ However, the second electron transfer required for catalytic turnover to produce H_2_ was several orders of magnitude slower than the first reduction step.^14^ The multi-electron nature of proton reduction therefore gives photo-generated TiO_2_-CB electrons time to undergo charge recombination and additional competing side reactions such as reduction of oxidised donor (AA) or oxidation products of the SED thereby limiting the overall efficiency of the system.

## Conclusions

In summary, we report the use of DPP-sensitised TiO_2_ for the assembly of a molecule-based DSP system for light-driven H_2_ generation in water without the need for a precious metal-containing component. Five novel DPP dyes bearing different side chains and a phosphonic acid-anchoring group, for robust immobilisation on metal oxide semiconductors, have been synthesised and are reported. The dyes exhibit strong light absorption over a wide range of the visible light spectrum (*λ* = 400 to 575 nm) and operate as efficient photosensitisers when adsorbed on TiO_2_. We demonstrate preliminary structure–activity relationships between the DPP chromophore modifications and the solar-driven H_2_ evolution performances of the dye|TiO_2_|catalyst systems. Changing energetic parameters such as broader light-harvesting range and push–pull design architecture by adding of a conjugated thiophene or an electron rich unit, as in **DPP2** or **DPP5**, was revealed to be beneficial for the H_2_ evolution performances (*i.e.* TOF and TON) as long as they allow for efficient electron injection and dye regeneration. In parallel, we confirmed that tuning non-energetic parameters (*e.g.* steric hindrance, position and nature of the solubilising side chains) plays a decisive role on the dye organisation at the TiO_2_ surface and the electronic communication with the media's components (**DPP4**). It is also evident that kinetic parameters (*e.g.* the lifetime of the charge-separated state) need to be considered and should be adapted in line with the catalyst kinetics to allow for sufficient time to perform the two-electron H_2_ evolution reaction. The performance of the dye in DSP systems does ultimately also depend on the pH, SED, chemical catalyst and mechanistic details, which implies that the comparison between two dyes' activity should be taken with caution. Nevertheless, the present study provides the basis for further studies to more fully rationalise dye design and structure–activity relationships in the future.

Compared to previous systems with the phosphonated Ru dye **RuP**, the DPP-systems can absorb light at higher wavelengths (up to 575 nm) and match the performance of the Ru dye in terms of stability and turnover numbers.^21,22,45,52^ It is promising that despite faster recombination kinetics of the DPP cations, reasonably efficient dye regeneration by AA is still observed. The compatibility of DPP with a hydrogenase demonstrates its biocompatibility and replacing the molecular catalysts by Pt demonstrates that DPP-based dyes outperform **RuP** in this system, which shows much scope for further development. We have therefore established phosphonated DPP dyes as an excellent alternative to precious metal-containing dyes in aqueous DSP schemes. The five DPP dyes studied herein are first-generation dyes and not yet fully optimised, leaving room for further tuning through core and side chain engineering to improve light absorption, charge separation and regeneration yields. DPP chromophores have therefore great potential in DSP and, more widely, in aqueous photocatalysis.
