# Supplementary material for: Solar H2 evolution in water with modified diketopyrrolopyrrole dyes immobilised on molecular Co and Ni catalyst–TiO2 hybrids
Source: Chem Sci. 2017 Feb 3;8(4):3070–9. doi: 10.1039/c6sc05219c (PMC5380916; doi:10.1039/c6sc05219c)
Supplement: Supplementary file 1 [file SC-008-C6SC05219C-s001.pdf]

Electronic Supplementary Material (ESI) for Chemical Science.
This journal is © The Royal Society of Chemistry 2017

Electronic Supporting Information

Solar H_2 evolution in water with modified diketopyrrolopyrrole dyes immobilised on molecular Co and Ni catalyst-TiO_2 hybrids

Julien Warnan[a‡], Janina Willkomm[a‡], Jamues N. Ng[a], Robert Godin[b], Sebastian Prantl[b], James R. Durrant[b] and Erwin Reisner[a,*]

[a]Christian Doppler Laboratory for Sustainable SynGas Chemistry, Department of Chemistry, University of Cambridge, Lensfield Road, Cambridge, CB2 1EW, UK.

[b] Department of Chemistry, Imperial College London, Exhibition Road, London, SW7 2AZ, UK.

*E-mail: reisner@ch.cam.ac.uk; Web: http://www-reisner.ch.cam.ac.uk

Experimental Section pages S2 – S16

Supporting Tables S1 – S10 pages S17 – S21

Supporting Figures S1 – S11 pages S22 – S27

Supporting References page S27

Experimental Section

Materials and methods

Chemicals for analytical measurements were of the highest available purity. Aeroxide P25 TiO_2 particles (anatase/rutile: 8/2 mixture, average particle size = 21 nm) were provided by Evonik Industries and ZrO_2 nanoparticles (99.9%, 20−30 nm) were obtained from Skyspring Nanomaterials Inc. The TiO_2 paste (Ti-Nanoxide T/SP, 100% anatase) was purchased from Solaronix. ITO sheets were purchased from Vision Tel System Ltd (40 x 50 cm^2, 30 Ω sq^{-1}). ITO nanopowder was obtained from Sigma Aldrich (diameter < 40 nm; BET = 27 m^2 g^{-1}; 90% In_2O_3, 10% SnO_2). $(Et_3NH)[CoCl(dmgH)_2(4\text{-}pyPO_3H)]$ (**CoP**), $[Ni(P^{Ph}{}_2N^{PhCH2PO(OH)2}{}_2)_2]Br_2{\cdot}HBr$ (**NiP**), [Ru(2,2'-bipyridine)$_2$(2,2'-bipyridine-4,4'-bisphosphonic acid)]Br_2 (**RuP**), platinised TiO_2 and compounds **5** and **6** were synthesised according to reported procedures.[1-5] The [NiFeSe]-hydrogenase (H_2ase) from *Desulfomicrobium baculatum* (*Dmb*) was obtained from the laboratory of Dr. Fontecilla-Camps (CRNS Grenoble, France) and has a specific activity of 2115 µmol H_2 min^{-1}. Microwave-assisted reactions were performed in a Biotage initiator. Column chromatography was carried out with silica gel 60 (0.040-0.063 mm mesh) from Material Harvest. Methanol (MeOH), toluene, tetrahydrofuran (THF), triethylamine (Et_3N) and dichloromethane (DCM) were distilled on sodium (THF & toluene) or calcium hydride (Et_3N, MeOH & DCM) before use.

Physical characterisation

^1H, ^{13}C, ^{31}P NMR spectra were recorded on a Bruker DPX-400 MHz or a Bruker 500 MHz DCH cryoprobe spectrometer at room temperature. Chemical shifts are given in ppm and coupling constants in Hz. Chemical shifts for ^1H NMR spectra are referenced relative to residual protium in the deuterated solvent ($CHCl_3$: δ_H = 7.26 ppm for ^1H NMR and δ_C = 77.16 ppm for ^{13}C NMR; CD_2Cl_2: δ_H = 5.32 ppm for ^1H NMR and δ_C = 53.84 ppm for ^{13}C NMR; MeOD: δ_H = 3.31 ppm for ^1H NMR and δ_C = 49.00 ppm for ^{13}C NMR; DMSO-d_6: δ_H = 2.50 ppm for ^1H NMR and δ_C = 39.52 ppm for ^{13}C NMR). High resolution-mass spectra were recorded using a ThermoScientific Orbitrap Classic mass spectrometer. UV-vis spectra were collected using a Varian Cary 50 Bio UV-vis spectrometer. For solution spectra, a quartz cuvette (Hellma, 1 cm path length) was used. Emission spectra were recorded using a spectrofluorometer (FS5 Spectrofluorometer, Edinburgh Instrument). A quartz cuvette with a path length of 1 cm was used (Starna Scientific) and DPP dyes and **RuP** were excited at 460 and 450 nm, respectively. Attenuated total reflectance FT-IR spectra of the compounds were recorded on a Nicolet iS50 spectrometer. Elemental analysis was carried out by the Microanalysis Service of the Department of Chemistry, University of Cambridge, using a Perkin-Elmer 240 Elemental Analyser.

Preparation of mesoporous ITO electrodes

ITO-coated glass slides (1 x 2 cm^2) were cleaned by immersing them in a solution of distilled water, ammonia (25%) and hydrogen peroxide (30% w/v) in the ratio of (5:1:1 v/v) and heating at 70 °C for 30 min. The ITO-coated glass slides were sonicated in distilled water and dried at room temperature. An ITO suspension (20 wt% of ITO nanopowder in 5 M acetic acid in ethanol) was doctor-bladed onto the cleaned ITO glass slides with a circular area (0.28 cm^2) using Scotch® tape (3M) as spacers to prepare the mesostructured coating. The resulting ITO slides with the mesoporous ITO film (thickness approximately 3 μm)[6] were left to dry in air before removing the tapes and then annealed using a Carbolite furnace under atmospheric conditions using the following temperature program: heating from 25 °C to 350 °C (5 °C min^{-1}), holding at 350 °C for 20 min before slowly cooling down to room temperature in the furnace chamber. The mesoporous ITO electrodes were cleaned using an ozone cleaner (BioForce Nanoscience) for 15 min before soaking them into 1 mM solution of each dye in THF (except for **RuP**, 1 mM in H_2O) for 16 h. Subsequently, the electrodes were washed with ethanol and were kept in the dark before use.

Preparation of dye-sensitised TiO_2 films

Glass slides (2 x 2 cm^2) were cleaned by successively immersing the electrodes for 10 min in a solution of distilled soapy water, distilled water and then ethanol while sonicating. TiO_2 paste was slot-coated onto glass with a rectangular area (1 x 2 cm) using Scotch® tape (3M) as spacers. After removing the tape, the slides were sintered using a Carbolite furnace under atmospheric conditions using a previously published heating ramp.[7] The thickness of the resulting TiO_2 layers was estimated around 6 μm by scanning electron microscopy. The dyes were immobilised by soaking the electrodes into a DPP solution in THF (0.25 mM) or a **RuP** solution in H_2O (0.25 mM) for 16 h. Subsequently, the electrodes were washed with THF or H_2O and kept in the dark before use.

Electrochemical measurements

A three-electrode electrochemical setup was used to determine the redox potential for the DPP and **RuP** dyes. Tetrabutylammonium tetrafluoroborate ($TBABF_4$, 0.1 M, ≥99.0%, Sigma, recrystallised from water and dried overnight at 80 °C under vacuum) in acetonitrile was used as an electrolyte solution and was purged with N_2 for 15 min before each experiment. The cell employed a dye-sensitised mesoporous ITO working, a platinum mesh counter and a silver wire coated with AgCl as a pseudo-reference

electrode. The reference potential was calibrated by adding ferrocene (Fc) as internal standard at the end of the experiment. The applied potentials were subsequently referenced versus NHE by addition of 0.63 V (Fc^+/Fc = +0.63 V vs NHE).[8] All cyclic voltammetry experiments were performed at room temperature at a scan rate of 50 mV s^{-1} using an IviumStat potentiostat.

Preparation of SED solutions

Triethanolamine (TEOA) hydrochloride was used to prepare the TEOA buffer solution (0.1 M, pH 7). Ascorbic acid (AA) solutions (0.1 M, pH 4.5) were freshly prepared for each experiment from L-ascorbic acid. The pH of each solution was adjusted with an aqueous NaOH solution. The final pH value was confirmed after diluting the SED solutions to the final concentration of 0.1 M. For enzyme experiments, 2-(N-morpholino)ethanesulfonic acid (MES, 0.1 M) was added to the AA solution and the pH adjusted to 6.

Quantification of DPP1 and DPP4 loading on TiO_2 nanoparticles

2.5 mg of TiO_2 nanoparticles were dispersed in a TEOA or AA SED solution via sonication for 10 min, followed by addition of **DPP1** or **DPP4** (0.05 or 0.25 µmol; 1 mM in THF). The mixture (total volume of 3 mL) was stirred for 10 min, centrifuged (8000 rpm, 10 min) and the UV-vis spectra of the supernatant recorded after passing it through a syringe filter (0.2 µm membrane). The amount of dye attached to TiO_2 was determined by comparing the UV-vis spectrum of the dye before and after addition of TiO_2.

Photocatalytic experiments

TiO_2 (2.5 mg) nanoparticles were dispersed in a SED solution (2.95 mL–V_{cat}) in the photoreactor *via* sonication for 10 min and a solution containing the H_2 evolution catalyst was subsequently added (0.01–0.2 µmol, 10–200 µL (= V_{cat}) of a 1 mM **CoP** or **NiP** solution in H_2O or methanol, respectively). After stirring the resulting suspension for 10 min in air, **RuP** (0.05 µmol, 50 µL of a 1 mM solution in water H_2O) or DPP (0.05 µmol, 50 µL of a 1 mM solution in THF) was added. In the case of TiO_2-Pt, pre-platinised TiO_2 (2.5 mg) was dispersed in a SED solution (2.95 mL) in the photoreactor *via* sonication for 10 min. **RuP** (0.05 µmol, 50 µL of a 1 mM solution in water H_2O) or **DPP2** (0.05 µmol, 50 µL of a 1 mM solution in THF) was then added. For experiments with hydrogenase, **RuP** (0.05 µmol, 50 µL of a 1 mM solution in water H_2O) or **DPP2** (0.05 µmol, 50 µL of a 1 mM solution in THF) was added first and the resulting suspension purged with nitrogen for 10 min before addition of the enzyme (50 pmol, 16.7 µL of a 3 µM pH 7 TEOA solution).

The photoreactor was sealed, kept in the dark after addition of the dye and purged with N_2 containing 2% methane (as internal standard for gas chromatography analysis) for 10 min. The total volume of the photocatalyst suspension was 3 mL leaving a gas headspace

volume of 4.84 mL. A LOT solar light simulator (1000 W Xenon lamp) irradiated an area of approximately 3.3 cm^2 of the stirred dye-sensitised photocatalyst suspension. The light source was equipped with an AM 1.5G filter, a water filter to remove IR irradiation and a 420 nm cut-off filter to avoid direct UV band gap photo-excitation of TiO_2. The light simulator was calibrated to 1 sun irradiation intensity (100 mW cm^{-2}). Samples were kept at 25 °C with a temperature controlled water bath and stirred during the course of the reaction.

Gas chromatography was used to analyse the headspace of the photoreactor in regular time intervals. The gas chromatograph (GC, Agilent 7890A Series) was equipped with a 5 Å molecular sieve column (held at 45 °C) and a thermal conductivity detector. Nitrogen was used as carrier gas at a flow rate of approximately 3 mL min^{-1}. The GC was calibrated in regular intervals to determine the response factor of hydrogen to the internal standard methane. All experiments were performed at least in triplicate (unless otherwise noted) and the mean values and standard deviations (error) are reported (see below for statistical analysis). A minimum of 10% error was assumed for all experiments.

$$\mathrm{TON_{cat}} = \frac{n(\mathrm{H_2})}{n(\mathrm{catalyst})} \qquad \mathrm{TON_{dye}} = \frac{n(\mathrm{H_2})}{n(\mathrm{dye})} \cdot 2$$

where TON = turnover number, n(H_2) = moles of photo-generated H_2, n(catalyst) = moles of catalyst and n(dye) = moles of dye.

$$\mathrm{TOF_{cat}}\,(1\mathrm{h}) = \frac{n(\mathrm{H_2})_{1\mathrm{h}}}{n(\mathrm{catalyst}) \cdot t} \qquad \mathrm{TOF_{dye}}\,(1\mathrm{h}) = \frac{n(\mathrm{H_2})_{1\mathrm{h}}}{n(\mathrm{dye}) \cdot t} \cdot 2$$

Where TOF (1h) = turnover frequency after 1h of irradiation, n$(H_2)_{1h}$ = moles of photo-generated H_2 after 1 h of irradiation, n(catalyst) = moles of catalyst, n(dye) = moles of dye and t = time of irradiation (1 h).

$$x_u = \sum_i \frac{x_i}{n} \qquad \sigma = \sqrt{\sum_i \frac{(x_i - x_u)^2}{(n-1)}}$$

where x_u = unweighted mean value, x_i = observations, n = number of observations and σ = standard deviation.

Determination of external quantum efficiency (EQE)

The EQE was determined for **RuP**|TiO_2|**NiP** and **DPP2**|TiO_2|**NiP** at the following wavelengths: λ = 400, 450, 475, 500, 550 and 575 nm. A LED light source (Ivium Modulight) was used and the light intensity (3.05 or 3.13 mW cm^{-2}, see Table S7) measured with a radiometric detector coupled to an optical power meter (ILT 1400 radio and photometer). The irradiated area was 0.283 cm^2. Samples were prepared as described above using TiO_2 (2.5 mg), **NiP** (0.025 µmol) and **RuP** or **DPP2** (0.05 µmol) in a total volume of 3 mL (AA, 0.1

M). An airtight quartz cuvette was used as photoreactor, filled with the photocatalytic suspension and sealed with a rubber septum. Samples were purged with N_2 (including 2% methane as internal standard) for 10 min prior to the measurements and samples (40 µL) of the remaining headspace (0.89 mL) of the cuvette were analysed *via* GC after 2 h of irradiation. Experiments were run at least in duplicate and the EQE was determined using the following equation:

$$EQE(\%) = \frac{n(\mathrm{H_2}) \cdot N_\mathrm{A} \cdot h \cdot c \cdot 2}{\lambda \cdot t \cdot I \cdot A} \cdot 100$$

where $n(\mathrm{H_2})$ = moles of photo-generated H_2 [mol], N_A = Avogadro constant [$\mathrm{mol^{-1}}$], h = Planck constant [J s]; c = speed of light [$\mathrm{m\ s^{-1}}$], λ = wavelength [m], t = irradiation time [s], I = light intensity [$\mathrm{W\ m^{-2}}$] and A = irradiated area [$\mathrm{m^2}$].

TAS measurements

Microsecond to second transient absorption decays were acquired in transmission mode. The experimental setup used a Nd:YAG laser (OPOTEK Opolette 355 II, 7 ns pulse width) as the tunable excitation source. The excitation fluences at 500 nm for experiments with the **DPP**-sensitised TiO_2 films were adjusted between 160 and 200 µJ $\mathrm{cm^{-2}}$ in order to obtain the same number of absorbed photons in each case. For **RuP** samples, excitation fluences between 30 and 680 µJ $\mathrm{cm^{-2}}$ were used and no fluence dependence on the kinetics was observed. Laser repetition rates were typically near 1 Hz. A quartz halogen lamp was used as the probe light source. Combination of optical filters and monochromators were used to minimise short wavelength irradiation of the sample and spectrally separate the desired signal from fluorescence and laser scatter. Time-resolved intensity data was collected with a Si photodiode (Hamamatsu S3071). Data < 1 ms was recorded by an oscilloscope after passing through amplifying electronics (Costronics) while data > 1 ms was simultaneously recorded on a National Instrument DAQ card. A few hundred laser pulses were averaged to obtain the kinetic traces. Samples were purged with inert gas (N_2 or argon) before acquisitions. Data was acquired and processed using home-built software written in the Labview environment.

Calculation of regeneration efficiencies

In order to estimate the overall regeneration efficiencies of each dye, we obtained the mean lifetime from stretched exponential fits (Fig. S11) according to:

$$\langle \tau \rangle = \frac{1}{\beta} \tau_{kww} \Gamma\left(\frac{1}{\beta}\right)$$

Here, Γ is the mathematical gamma function.

In order to compare the decays with and without AA and take into account the regeneration that took place on timescales shorter than our experimental setup could measure, we fixed the amplitude A in the stretched exponential expression to that obtained from the fitting in H_2O when fitting the decay in AA. In this fashion, we normalise the decays to the same amount of photogenerated dye cation, as expected under the assumption that the injection yield has not been influenced by the addition AA. Simplifying to first order competitive kinetics, the quantum yield of generation is then calculated as:

$$QY_{reg} = \frac{k_{reg}}{k_{reg} + k_{CS}}; k_{AA} = k_{reg} + k_{CS}$$

$$QY_{reg} = \frac{k_{AA} - k_{CS}}{k_{AA}};\ k_{AA} = \frac{1}{\langle\tau\rangle_{AA}}, k_{CS} = \frac{1}{\langle\tau\rangle_{H_2O}}$$

We calculate regeneration efficiencies of 52% (**DPP1**), 94% (**DPP2**), 88% (**DPP5**), 100% (**RuP**).

Synthesis and characterisation of DPP dyes.

General procedure for Suzuki-Miyaura cross-coupling: Synthesis of **7**, **8** and **10**

Compound **1** or **2** (6.3×10^{-4} mol), [Pd(PPh_3)$_4$] (2.1×10^{-5} mol), and sodium carbonate (6.3×10^{-3} mol) were solubilised in a mixture of THF (8 mL) and H_2O (4 mL) at room temperature under a N_2 atmosphere. The reaction mixture was heated at 45 °C for 0.5 h, then a solution of the corresponding compound **3** or **4** (6.9×10^{-4} mol) in THF (8 mL) was added. The temperature was increased to 80 °C and maintained for 16 h. Once cooled to room temperature, water (20 mL) was poured to the reaction mixture and the crude product extracted with DCM (20 mL x2). The organic layer was then washed with water, brine, dried on $MgSO_4$, filtered and concentrated under vacuum.

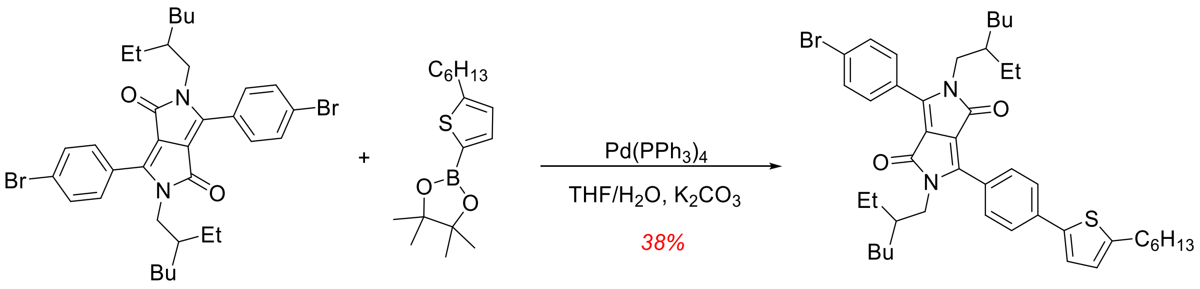

1 **3** **7**

Compound **7** (from reaction of **1** and **3**): The crude product was purified by silica gel column chromatography (eluted with DCM and hexane (8/2)) in 38% yield. The product **7** was obtained from the 2nd fraction and isolated as a dark red oil. ^{1}H NMR ($CDCl_3$, 400 MHz): δ_H (ppm)= 7.80 (d, J = 8.5 Hz, 2H), 7.68 (d, J = 8.5 Hz, 2H), 7.65 (s, 4H), 7.24 (d, J = 3.6 Hz,

1H), 6.78 (d, J = 3.6 Hz, 1H), 3.82-3.67 (m, 4H), 2.84 (t, J = 7.7 Hz, 2H), 1.71 (m, 2H), 1.60-1.46 (bm, 2H), 1.44-1.25 (bm, 6H), 1.23-0.99 (bm, 16H), 0.90 (m, 3H), 0.81-0.73 (m, 6H), 0.72 (t, J = 7.4 Hz, 6H); ^{13}C NMR ($CDCl_3$, 100 MHz): δ_C (ppm)= 163.0, 162.7, 149.0, 147.6, 146.9, 140.6, 137.5, 132.3, 130.2, 129.4, 127.6, 126.7 125.6, 125.5 (×2), 124.2, 110.3, 109.8, 45.3, 45.1, 38.7, 38.6, 31.7, 30.5, 28.9, 28.4, 23.9, 23.0, 22.7, 14.2, 14.1, 10.6; HRMS (+ESI, m/z): calcd. for $C_{44}H_{58}O_2N_2{}^{79}Br_1{}^{32}S_1$ $[M+H]^+$: 757.3392; found, 757.3397.

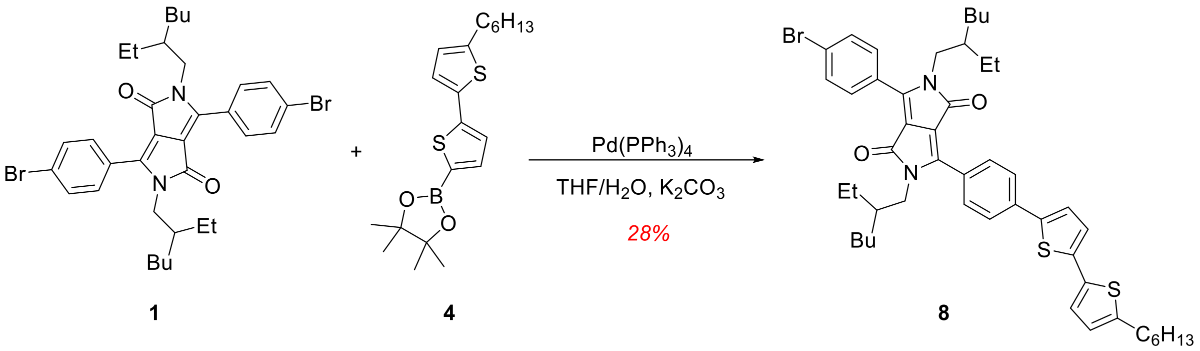

1 **4** **8**

Compound **8** (from reaction of **1** and **4**): The crude product was purified by silica gel column chromatography (eluted with DCM and MeOH (98/2)) in 28% yield. ^{1}H NMR ($CDCl_3$, 400 MHz): δ_H (ppm)= 7.83 (d, J = 8.5 Hz, 2H), 7.72 (d, J = 8.5 Hz, 2H), 7.66 (s, 4H), 7.32 (d, J = 3.8 Hz, 1H), 7.11 (d, J = 3.8 Hz, 1H), 7.05 (d, J = 3.6 Hz, 1H), 6.71 (d, J = 3.5 Hz, 1H), 3.80-3.70 (m, 4H), 2.81 (t, J = 7.3 Hz, 2H), 1.70 (m, 2H), 1.43-1.00 (bm, 24H), 0.90 (m, 3H), 0.79 (m, 6H), 0.73 (m, 6H); ^{13}C NMR ($CDCl_3$, 100 MHz): δ_C (ppm)= 163.2, 163.0, 149.3, 147.7, 147.5, 140.6, 137.8, 132.2, 131.1, 130.1, 127.1, 126.2, 125.6, 125.5, 124.3, 110.1, 109.6, 72.0, 71.9, 70.7, 69.1, 59.3, 59.2, 42.5, 42.4, 31.7, 30.5, 28.9, 22.7, 14.2; HRMS (+ESI, m/z): calcd. for $C_{48}H_{60}O_2N_2{}^{79}Br_1{}^{32}S_2$ $[M+H]^+$: 839.3274; found, 839.3266.

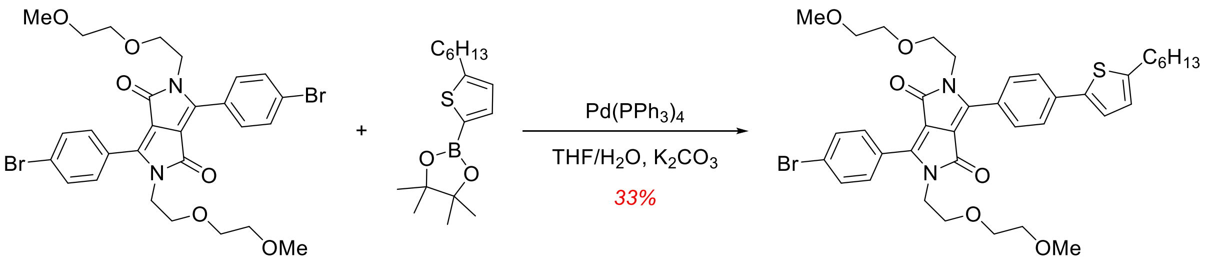

2 **3** **10**

Compound **10** (from reaction of **2** and **3**): The crude product was purified by silicagel column chromatography (eluted with DCM and hexane (7/3)) in 33% yield. ^{1}H NMR ($CDCl_3$, 400 MHz): ^{13}C NMR ($CDCl_3$, 100 MHz): δ_H (ppm)= 8.03 (d, J = 8.6 Hz, 2H), 7.92 (d, J = 8.6 Hz, 2H), 7.69 (d, J = 8.6 Hz, 2H), 7.64 (d, J = 8.6 Hz, 2H), 7.24 (d, J = 3.6 Hz, 1H), 6.78 (d, J = 3.6 Hz, 1H), 3.99 (t, J = 5.5 Hz, 2H), 3.93 (t, J = 5.3 Hz, 2H), 3.80-3.73 (m, 4H), 3.55 (m, 4H), 3.46 (m, 4H), 3.34 (s, 6H), 2.84 (t, J = 7.6 Hz, 2H), 1.71 (m, 2H), 1.45-1.29 (m, 6H), 0.90 (m, 3H); δ_C (ppm)= 162.8, 162.6, 148.6, 146.9, 146.1, 141.0, 138.8, 136.7, 134.4,

132.1, 130.1, 129.3, 127.4, 127.0, 125.4 (×2),125.0, 124.9, 124.0, 123.8, 110.2, 109.7, 45.2, 45.0, 38.6, 38.5, 31.6, 30.3, 30.2, 29.7, 28.7, 28.2, 23.7, 22.9, 22.6, 14.1, 13.9, 10.4; HRMS (+ESI, m/z): calcd. for $C_{38}H_{46}O_6N_2{}^{79}Br_1{}^{32}S_1$ $[M+H]^+$: 737.2288; found, 737.2260.

General procedure for Stille cross-coupling: Synthesis **9** & **11**

Compound **1** (2.3×10^{-4} mol), $[Pd(PPh_3)_4]$ (1.2×10^{-5} mol) and the corresponding compound **5** or **6** (2.6×10^{-4} mol) were solubilised in dry toluene (7.5 mL) under a N_2 atmosphere. The reaction mixture was degassed by N_2 bubbling under sonication for 15 min before the Schlenk tube was dipped into a preheated oil bath at 80 °C. The solution was then heated at 80 °C for 16 h. Once cooled to room temperature, water (15 mL) was added to the reaction mixture and the crude product extracted with dichloromethane (20 mL ×2). The organic layer was the washed with water, brine, then dried on $MgSO_4$, filtered and concentrated under vacuum.

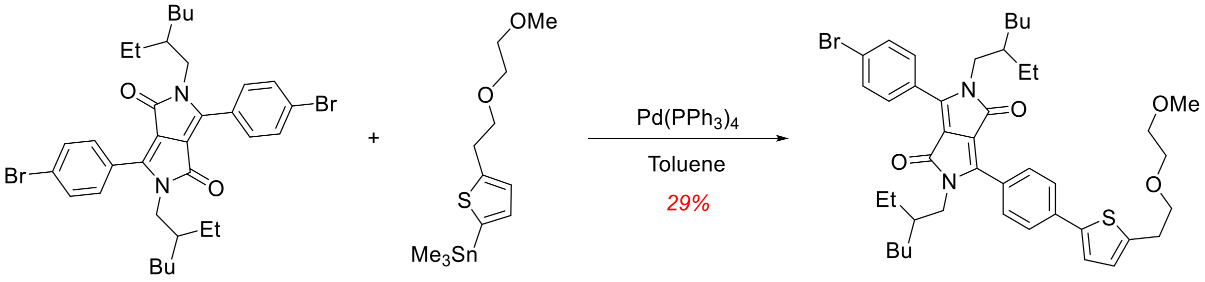

1 **5** **9**

Compound **9** (from reaction of **1** and **5**): The crude product was purified by silicagel column chromatography (eluted with DCM and MeOH (99/1)) in 29% yield. The product **9** was obtained from the 2nd fraction and isolated as a dark red oil. ^1H NMR ($CDCl_3$, 400 MHz): δ_H (ppm)= 7.80 (d, J = 8.4 Hz, 2H), 7.68 (d, J = 8.4 Hz, 2H), 7.64 (s, 4H), 7.24 (d, J = 3.6 Hz, 1H), 6.85 (d, J = 3.5 Hz, 1H), 3.80-3.58 (bm, 6H), 3.66 (m, 2H), 3.58 (m, 2H), 3.41 (s, 3H), 3.14 (t, J = 6.7 Hz, 2H), 1.50 (m, 2H), 1.23-0.99 (bm, 16H), 0.81-0.78 (m, 6H), 0.72 (t, J = 7.4 Hz, 6H); ^{13}C NMR ($CDCl_3$, 100 MHz): δ_C (ppm)= 162.9, 162.7, 148.9, 147.0, 142.8, 141.4, 137.4, 132.3, 130.2, 129.4, 127.6, 126.9, 126.7, 125.6, 124.2, 110.3, 109.8, 72.1, 71.9, 70.5, 59.3, 45.3, 45.1, 38.7, 38.6, 31.0, 30.4, 28.4, 23.9, 23.0, 14.1, 10.6; HRMS (+ESI, m/z): calcd. for $C_{43}H_{56}O_4N_2{}^{79}Br_1{}^{32}S_1$ $[M+H]^+$: 775.3179; found, 775.3144.

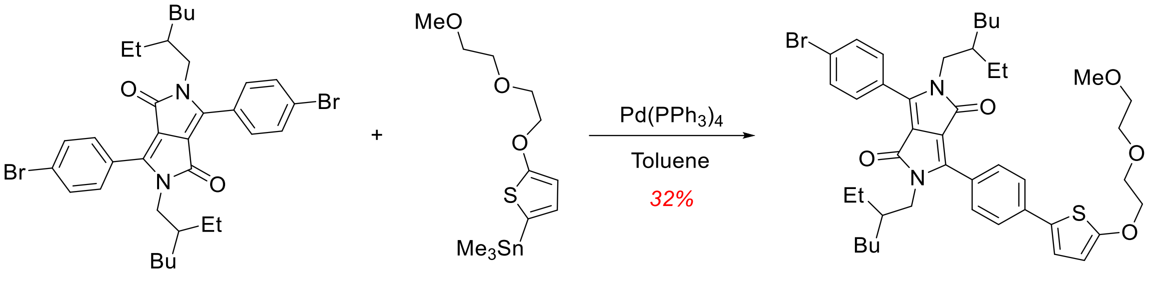

1 **6** **11**

Compound **11** (from reaction of **1** and **6**): The crude product was purified by silica gel column chromatography (eluted with DCM and MeOH (98/2)) and a second column (using hexane and ethyl acetate (7/3)) in 32% yield. The product **11** was isolated as a dark red oil. ^{1}H NMR ($CDCl_3$, 400 MHz): δ_H (ppm)= 7.79 (d, J = 8.4 Hz, 2H), 7.65 (s, 4H), 7.60 (d, J = 8.4 Hz, 2H), 7.09 (d, J = 4.0 Hz, 1H), 6.26 (d, J = 4.0 Hz, 1H), 4.26 (t, J = 4.8 Hz, 2H), 3.87 (t, J = 4.4 Hz, 2H), 3.79-3.70 (m, 6H), 3.59 (m, 2H), 3.41 (s, 3H), 1.50 (m, 2H), 1.23-0.99 (bm, 16H), 0.81-0.78 (m, 6H), 0.72 (m, 6H); ^{13}C NMR ($CDCl_3$, 100 MHz): δ_C (ppm)= 166.1, 163.0, 162.6, 149.0, 146.8, 137.5, 132.2, 130.2, 129.5, 129.4, 127.6, 126.2, 125.4, 124.8, 122.4, 110.3, 109.6, 106.7, 73.2, 72.1, 71.0, 69.5, 59.3, 45.3, 45.1, 38.7, 38.6, 30.4, 29.8, 28.4, 23.9, 23.0, 14.1, 10.6; HRMS (+ESI, m/z): calcd. for $C_{43}H_{56}O_5N_2{}^{79}Br_1{}^{32}S_1$ $[M+H]^+$: 791.3058; found, 791.3088.

General procedure for Hirao cross-coupling: Synthesis **12** to **16**

Compound **12**, **13**, **14**, **15** or **16** (1.6×10^{-4} mol) and $[Pd(PPh_3)_4]$ (1.2×10^{-5} mol) were added to a microwave (MW) reactor and solubilised in dry THF (2.5 mL). The MW reactor was sealed under N_2 before dry Et_3N (2.0×10^{-4} mol) and freshly distilled $HPO(OEt)_2$ (1.7×10^{-4} mol) were added through the septum. The reaction mixture was then placed under MW irradiation for 0.5 h at 120 °C (resulting pressure = 2-3 bar). After cooling to room temperature, a white precipitate was observed. DCM (10 mL) and water (10 mL) were added and the aqueous layer was extracted twice before the combined organic layers were washed with water (15 mL) and brine (15 mL). The orange/red solution was then dried on Na_2SO_4, filtered and concentrated under vacuum.

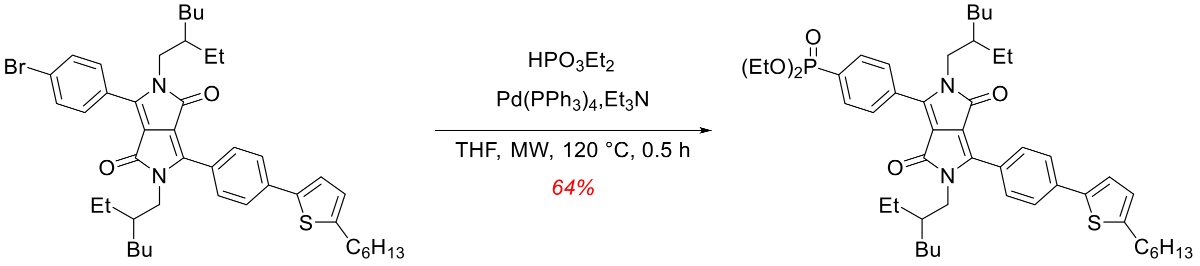

7 **12**

Compound **12** (from reaction of **7**): The crude product was purified by silica gel column chromatography (eluted with a gradient using ethyl acetate and hexane ratio from 7/3 to 9/1) in 64% yield. The product **12** was isolated as an orange solid. ^{1}H NMR ($CDCl_3$, 400 MHz): δ_H (ppm)= 7.95 (m, 2H), 7.87-7.83 (m, 2H), 7.82 (d, J = 8.4 Hz, 2H), 7.70 (d, J = 8.4 Hz, 2H), 7.25 (d, J = 3.5 Hz, 1H), 6.79 (d, J = 3.5 Hz, 1H), 4.25-4.00 (bm, 4H), 3.76 (m, 4H), 2.84 (t, J = 7.7 Hz, 2H), 1.71 (m, 2H), 1.50 (m, 2H), 1.47-1.25 (bm, 12H), 1.23-0.99 (bm, 16H), 0.90 (m, 3H), 0.80-0.66 (m, 12H); ^{13}C NMR ($CDCl_3$, 100 MHz): δ_C (ppm)= 162.9, 162.5, 149.5, 147.6, 146.4, 140.4, 137.5, 132.2, 132.1, 129.3, 128.5, 128.4, 126.4, 125.5, 125.4, 124.2, 110.8, 109.7, 62.4 (×2), 45.2, 45.0, 38.7, 38.5, 31.6, 30.4, 30.3, 29.7, 28.8, 28.2, 23.7, 23.6, 22.9, 22.8, 22.6, 16.4, 16.3, 14.1, 14.0, 13.9; ^{31}P NMR ($CDCl_3$, 202 MHz) δ_P (ppm)= 17.11; HRMS (+ESI, m/z): calcd. for $C_{48}H_{68}O_5N_2P_1{}^{32}S_1$ $[M+H]^+$: 815.4581; found, 815.4553.

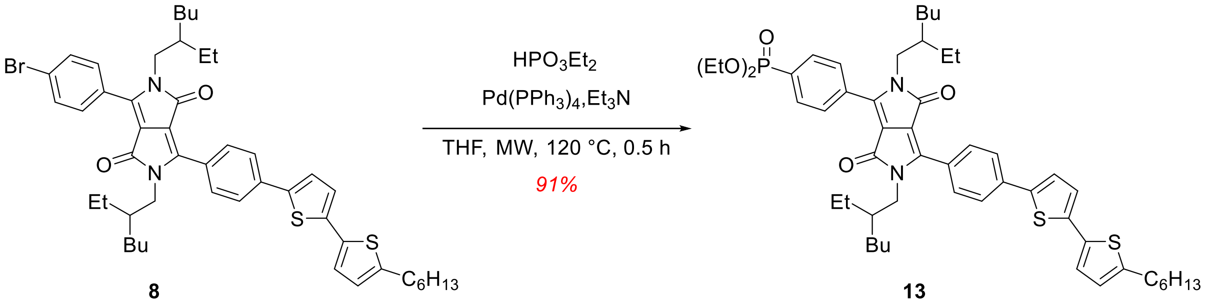

Compound **13** (from reaction of **8**): The crude product was purified by silica gel column chromatography (eluted with DCM and MeOH (95/5)) in 91% yield. The product **13** was isolated as an orange bright solid. ^{1}H NMR (CD_2Cl_2, 400 MHz): δ_H (ppm)= 7.97-7.76 (m, 6H), 7.75 (d, 2H), 7.39 (d, J = 3.8 Hz, 1H), 7.14 (d, J = 3.8 Hz, 1H), 7.08 (d, J = 3.6 Hz, 1H), 6.74 (d, J = 3.6 Hz, 1H), 4.12 (m, 4H), 3.76 (m, 4H), 2.82 (t, J = 7.6 Hz, 2H), 1.70 (m, 2H), 1.50 (m, 2H), 1.44-1.25 (m, 12H), 1.24-1.01 (bm, 16H), 0.90 (m, 3H), 0.79 (t, 6H), 0.76-0.67 (m, 6H); ^{13}C NMR ($CDCl_3$, 125 MHz): δ_C (ppm)= 163.1, 162.7, 149.9, 147.6, 146.9, 140.4, 137.8, 132.1, 132.0, 131.9, 131.7, 130.2, 130.0, 129.3, 129.2, 128.5, 128.4, 125.9, 125.5, 125.4, 124.2, 110.7, 109.5, 72.6, 71.8 (×2), 71.6, 70.6, 70.5, 70.3, 70.0, 68.9, 62.4, 62.3, 61.7, 59.1, 42.3, 31.9, 31.6, 30.3, 29.7, 29.4, 28.8, 26.1, 22.7, 22.6, 16.4, 16.3, 14.1; ^{31}P NMR ($CDCl_3$, 202 MHz) δ_P (ppm)= 17.25; HRMS (+ESI, m/z): calcd. for $C_{52}H_{70}O_5N_2P_1{}^{32}S_2$ $[M+H]^+$: 897.4458; found, 897.4422.

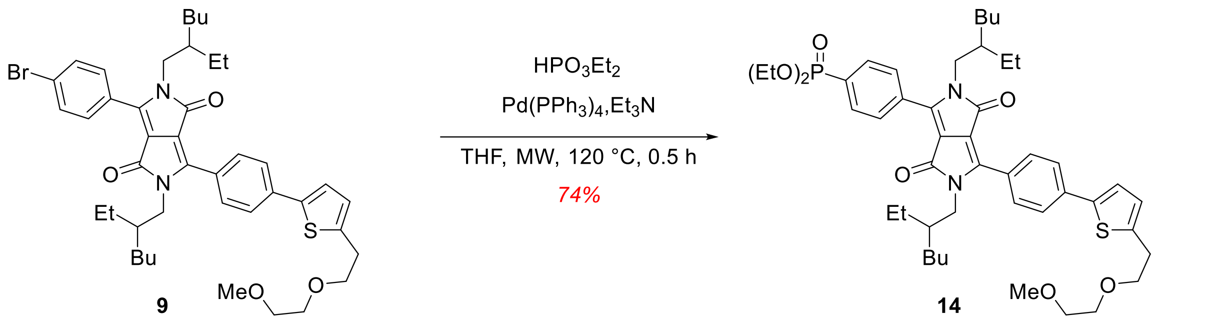

9 **14**

Compound **14** (from reaction of **9**): The crude product was purified by silica gel column chromatography (eluted with dichloromethane and methanol (96/4)) in 74% yield. The product **14** was isolated as a red powder. ^{1}H NMR (CD_2Cl_2, 400 MHz): δ_H (ppm)= 7.90 (m, 4H), 7.83 (d, J = 8.4 Hz, 2H), 7.74 (d, J = 8.4 Hz, 2H), 7.31 (d, J = 3.6 Hz, 1H), 6.89 (d, J = 3.6 Hz, 1H), 4.12 (m, 4H), 3.80-3.70 (bm, 6H), 3.62 (m, 2H), 3.53 (m, 2H), 3.35 (s, 3H), 3.11 (t, J = 6.7 Hz, 2H), 1.50 (m, 2H), 1.34 (t, J = 7.1 Hz, 6H) 1.23-1.00 (bm, 16H), 0.90 (t, J = 7.1 Hz, 6H), 0.75-0.66 (m, 6H; ^{13}C NMR (CD_2Cl_2, 100 MHz): δ_C (ppm)= 162.7, 162.6, 149.1, 146.6, 141.2, 139.1, 136.9, 134.6, 133.0, 132.3, 132.2, 131.0, 129.7, 128.8, 128.7, 127.4, 125.6, 125.5, 125.3, 124.3, 124.1, 111.1, 110.2, 62.6 (×2), 45.2, 45.0, 39.1, 38.9, 31.9, 31.8, 30.5, 30.4, 29.0, 28.6, 24.0, 23.9, 23.1 (×2), 22.9, 16.5, 16.4, 14.1, 14.0 (×2), 10.5, 10.4; ^{31}P NMR (CD_2Cl_2, 202 MHz) δ_P (ppm)= 16.59; HRMS (+ESI, m/z): calcd. for $C_{47}H_{66}O_7N_2P_1{}^{32}S_1$ $[M+H]^+$: 833.4323; found, 833.4295.

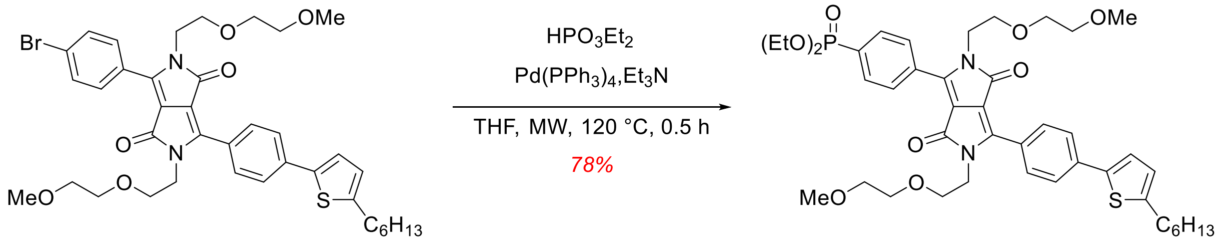

10 **15**

Compound **15** (from reaction of **10**): The crude was purified by silica gel column chromatography eluted with a mixture of DCM and MeOH (96/4) in 78% yield. The product **15** was isolated as an orange powder. ^{1}H NMR ($CDCl_3$, 400 MHz): δ_H (ppm)= 8.10 (m, 2H), 8.07 (d, J = 8.3 Hz, 2H), 7.94 (m, 2H), 7.69 (d, J = 8.3 Hz, 2H), 7.24 (d, J = 3.2 Hz, 1H), 6.78 (d, J = 3.2 Hz, 1H), 4.16 (m, 4H), 3.99 (m, 2H), 3.94 (m, 2H), 3.80-3.74 (m, 4H), 3.55 (m, 4H), 3.46 (m, 4H), 3.34 (s, 6H), 2.84 (t, J = 7.6 Hz, 2H), 1.72 (m, 2H), 1.45-1.29 (m, 12H), 0.90 (m, 3H); ^{13}C NMR ($CDCl_3$, 100 MHz): δ_C (ppm)= 163.1, 162.7, 149.8, 147.6, 146.9, 140.4, 137.5, 132.1, 131.7, 130.0, 129.3, 129.1, 128.5, 125.9, 125.5, 124.2, 110.7, 109.5, 71.8, 70.6, 68.9, 62.4, 59.1, 42.3, 31.5, 30.3, 28.8, 22.6, 16.5, 16.4, 14.1; ^{31}P NMR ($CDCl_3$, 202 MHz) δ_P (ppm)= 17.24; HRMS (+ESI, m/z): calcd. for $C_{42}H_{56}O_9N_2P_1{}^{32}S_1$ $[M+H]^+$: 795.3439; found, 795.3428

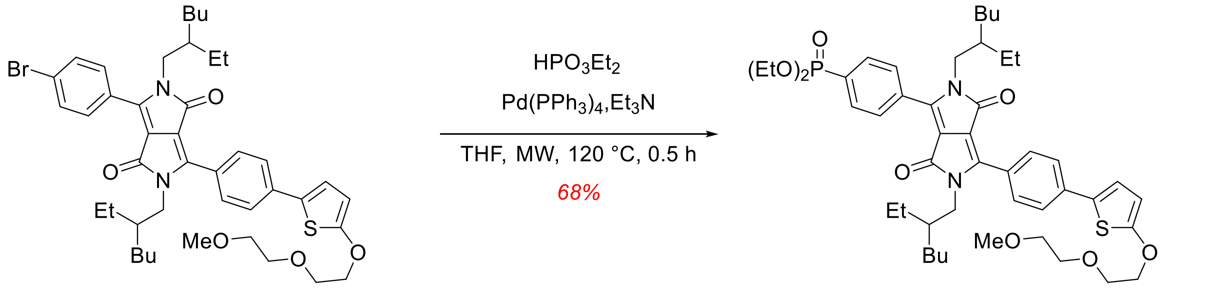

Compound **16** (from reaction of **11**): The crude product was isolated by silica gel column chromatography (first eluted with dichloromethane and methanol (98/2)), followed by another column using dichloromethane and acetone (96/4)) in 68% yield. The product **16** was isolated as an orange powder. ^{1}H NMR ($CDCl_3$, 400 MHz): δ_H (ppm)= 7.95 (m, 2H), 7.85 (m, 2H), 7.80 (d, J = 8.4 Hz, 2H), 7.61 (d, J = 8.4 Hz, 2H), 7.10 (d, J = 4.0 Hz, 1H), 6.27 (d, J = 4.0 Hz, 1H), 4.27 (t, J = 4.8 Hz, 2H), 4.16 (m, 4H), 3.88 (t, J = 4.6 Hz, 2H), 3.77-3.68 (m, 6H), 3.59 (m, 2H), 3.41 (s, 3H), 1.46 (m, 2H), 1.35 (t, J = 7.1 Hz, 6H), 1.23-0.99 (bm, 16H), 0.80-0.66 (m, 12H); ^{13}C NMR ($CDCl_3$, 125 MHz): δ_C (ppm)= 166.0, 162.9, 162.4, 149.5, 146.3, 137.6, 132.2, 132.1, 129.3, 129.0, 128.5, 128.4, 126.0, 124.7, 122.3, 110.8, 109.6, 106.6, 73.0, 71.9, 70.8, 69.4, 62.4 (×2), 59.1, 45.2, 45.0, 38.7, 38.4, 30.3, 29.7 28.2, 23.7, 23.6, 22.9, 22.8, 16.4, 16.3, 14.0, 13.9, 10.4, 10.3; ^{31}P NMR ($CDCl_3$, 202 MHz) δ_P (ppm)= 17.13; HRMS (+ESI, m/z): calcd. for $C_{47}H_{66}O_8N_2P_1{}^{32}S_1$ $[M+H]^+$: 849.4272; found, 849.4251.

General procedure for phosphonate ester hydrolysis: Synthesis **DPP1** to **DPP5**

The phosphonate ester compound (**12-16**; 6.4 $\times 10^{-5}$ mol) was placed in a dry round bottom flask and solubilised in dry DCM (3 mL) before bromotrimethylsilane (6.4 $\times 10^{-4}$ mol) was added dropwise. After stirring overnight at room temperature, the solvent was removed under vacuum. A mixture of MeOH (0.5 mL) and DCM (2.5 mL) was then added and the solution was stirred for 3 more hours. DCM (10 mL) and water (10 mL) were added and the aqueous layer was extracted until no coloration remains. The organic layer was then washed with water (10 mL) and brine (10 mL), dried over Na_2SO_4, filtered and concentrated under vacuum. The DPP dyes were obtained as pure products.

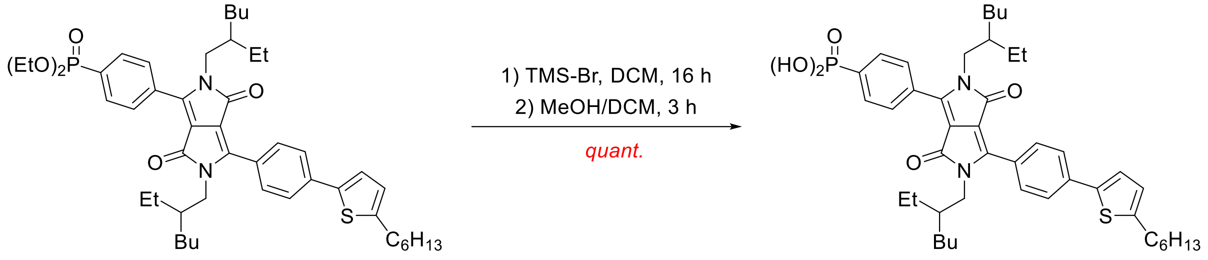

12 **DPP1**

DPP1 (from reaction of **12**): Quantitative yield. ^{1}H NMR (MeOD, 400 MHz): δ_H (ppm)= 7.98 (m, 2H), 7.84-7.73 (m, 6H), 7.35 (d, J = 3.4 Hz, 1H), 7.83 (d, J = 3.4 Hz, 1H), 3.81 (m, 4H), 2.86 (t, J = 7.5 Hz, 2H), 1.73 (m, 2H), 1.47-1.26 (bm, 8H), 1.23-1.03 (bm, 16H), 0.93 (m, 3H), 0.87-0.67 (m, 12H); ^{13}C NMR (MeOD, 125 MHz): δ_C (ppm)= 164.3, 164.2, 150.4, 148.5, 141.6, 138.9, 132.4, 132.3, 130.7, 129.0, 128.9, 127.9, 126.9, 126.2,125.5, 110.9, 110.7, 57.7, 57.5, 57.3, 46.1, 39.8, 39.6, 32.8, 32.8, 31.5, 31.4, 31.2, 29.9, 29.5, 29.4, 25.0 (×2), 23.9, 23.8, 23.7, 17.5, 17.3, 17.1, 14.4, 14.3 (×2), 10.9, 10.8; ^{31}P NMR (MeOD, 202 MHz) δ_P (ppm)= 9.90; FT-IR (ATR, σ/cm^{-1}): 3373, 2923, 1717, 1663, 1510, 1514, 1461, 1260, 1132, 1089, 1047. HRMS (+ESI, m/z): calcd. for $C_{44}H_{60}O_5N_2P_1{}^{32}S_1$ $[M+H]^+$: 759.3955; found, 759.3982. Anal. calcd. for $C_{44}H_{58}N_2O_5P_1S_1Na_1$ + $2H_2O$: C, 64.69; H, 7.65; N, 3.43; P, 3.79 found: C, 64.78; H, 7.58; N, 3.20; P, 3.53.

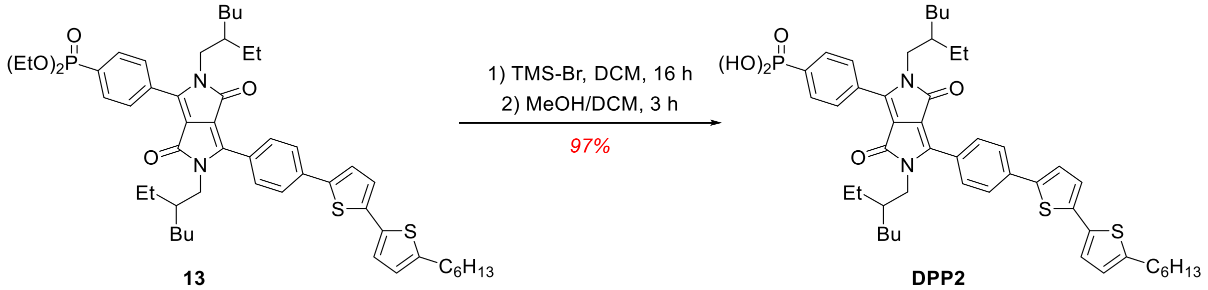

13 **DPP2**

DPP2 (from reaction of **13**): 97% yield. ^{1}H NMR (MeOD+CD_2Cl_2, 400 MHz): δ_H (ppm)= 7.87-7.72 (m, 6H), 7.65 (d, J = 8.4 Hz, 2H), 7.36 (d, J = 3.2 Hz, 1H), 7.03 (d, J = 3.7 Hz, 1H), 7.00 (d, J = 3.2 Hz, 1H), 6.70 (d, J = 3.3 Hz, 1H), 3.76 (m, 4H), 2.81 (t, J = 7.4 Hz, 2H), 1.71 (m, 2H), 1.48-1.26 (m, 8H), 1.22-1.02 (m, 16H), 0.93 (m, 3H); 0.84-0.66 (m, 12H); ^{13}C NMR ($CDCl_3$, 125 MHz): δ_C (ppm)= 162.4, 162.1, 149.0, 145.5, 140.8, 138.2, 136.0, 134.7, 131.4, 131.1, 129.5, 128.7, 128.6, 126.3, 124.7, 124.5, 124.4, 123.4, 109.4, 108.8, 44.4, 38.2, 37.9, 31.6 (×2), 30.3, 29.7, 28.9, 28.3, 28.2, 23.8, 23.7, 22.8 (2), 22.6, 14.1, 13.9 (2), 13.7, 10.4, 10.2; ^{31}P NMR (MeOD+CD_2Cl_2, 202 MHz) δ_P (ppm)= 13.35; FT-IR (ATR, σ/cm^{-1}): 2924, 1717, 1660, 1503, 1514, 1460, 1140, 1094. HRMS (+ESI, m/z): calcd. for $C_{48}H_{61}O_5N_2{}^{23}Na_1P_1{}^{32}S_2$ $[M+Na]^+$: 863.3652; found, 863.3650. Anal. calcd. for $C_{48}H_{59}N_2O_5P_1S_2Na_2$ + $2H_2O$: C, 62.59; H, 6.89; N, 3.04; P, 3.36 found: C, 63.00; H, 6.73; N, 2.89; P, 3.76.

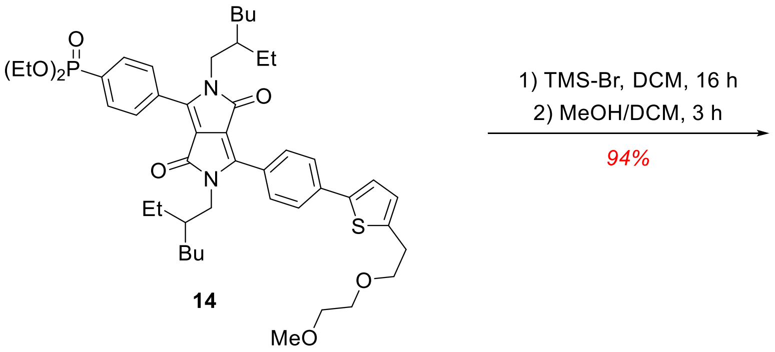

14 DPP3

DPP3 (from reaction of **14**): 94% yield. ^{1}H NMR (MeOD, 400 MHz): δ_H (ppm)= 7.79-7.58 (bm, 6H), 7.56-7.46 (bm, 2H), 7.17 (bs, 1H), 6.79 (bs, 1H), 3.79-3.63 (m, 10H), 3.59 (s, 3H), 3.08 (t, J = 6.5 Hz, 2H), 1.23 (bm, 2H), 1.19-0.98 (bm, 16H), 0.86-0.62 (m, 12H); ^{13}C NMR ($CDCl_3$, 125 MHz): δ_C (ppm)= 162.4, 162.1, 149.2, 147.8, 141.7, 141.3, 136.4, 131.4, 131.1, 129.4, 128.7, 128.6, 126.3, 124.5, 123.5, 109.5, 108.7, 71.9, 71.8, 70.3, 59.1, 44.4, 38.1, 37.9, 31.9, 30.8, 30.3, 29.7, 29.3, 28.3, 28.2, 23.8, 23.7, 22.8 (*2), 22.7, 14.1, 13.9 (*2), 10.4 (*2); ^{31}P NMR (MeOD, 202 MHz) δ_P (ppm)= 10.65; FT-IR (ATR, σ/cm^{-1}): 3371, 2925, 1664, 1508, 1460, 1087. HRMS (+ESI, m/z): calcd. for $C_{43}H_{57}O_7N_2{}^{23}Na_1P_1{}^{32}S_1$ $[M+Na]^+$: 799.3516; found, 799.3504.

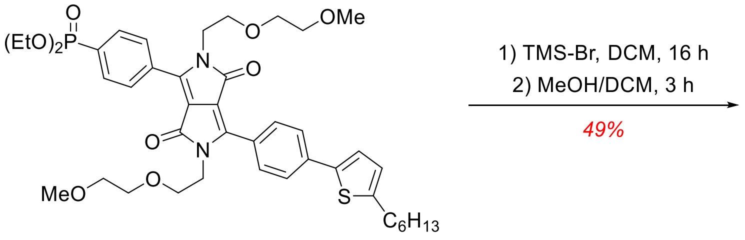

15 DPP4

DPP4 (from reaction of **15**): 49% yield. ^{1}H NMR (MeOD+CD_2Cl_2, 400 MHz): δ_H (ppm)= 8.06 (m, 6H), 7.75 (d, J = 7.6 Hz, 2H), 7.36 (d, J = 3.3 Hz, 1H), 6.84 (d, J = 3.3 Hz, 1H), 3.98 (m, 4H), 3.68 (m, 4H), 3.50 (m, 4H), 3.44 (m, 4H), 3.31 (s, 6H), 2.88 (t, J = 7.6 Hz, 2H), 1.74 (m, 2H), 1.48-1.26 (m, 6H), 0.94 (m, 3H); ^{13}C NMR: The limited solubility of the compound did not allow us to record a clear carbon NMR spectrum. ^{31}P NMR (THF-d_8, 202 MHz) δ_P (ppm)= 13.22; FT-IR (ATR, σ/cm^{-1}): 3428, 2919, 1664, 1508, 1083. HRMS (+ESI, m/z): calcd. for $C_{38}H_{47}O_9N_2{}^{23}Na_1P_1{}^{32}S_1$ $[M+Na]^+$: 761.2632; found, 761.2605.

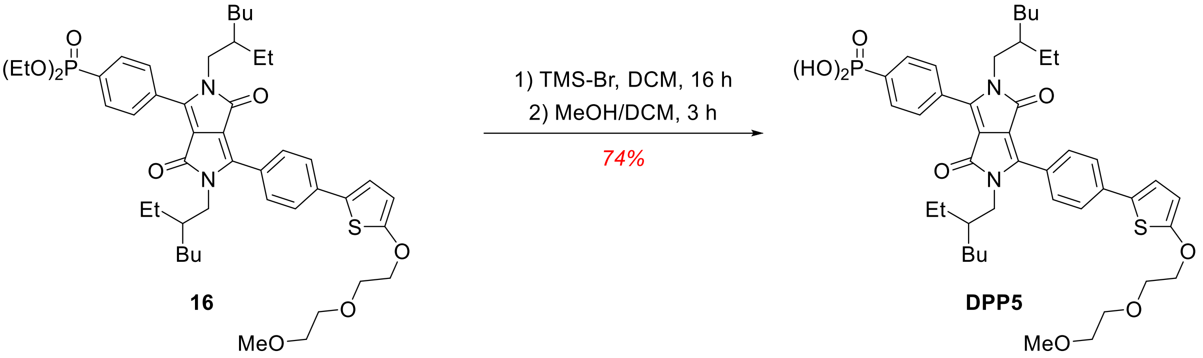

DPP5 (from reaction of **16**): 74% yield. ^{1}H NMR (DMSO, 500 MHz): δ_H (ppm)= 8.10-7.10 (bm, 9H), 6.35 (bs, 1H), 4.22 (bm, 2H), 3.77 (m, 2H), 3.61 (bm, 4H), 3.49 (m, 2H), 3.42 (m, 2H), 3.28 (bs, 3H), 1.26 (bm, 2H), 1.14-0.90 (bm, 16H), 0.80-0.52 (m, 12H); ^{13}C NMR (DMSO, 125 MHz): δ_C (ppm)= 165.4, 161.7, 161.5, 147.8, 136.4, 130.7, 129.6, 129.3, 127.9, 127.4, 125.4, 123.4, 108.4 (b), 106.4, 72.7, 72.2, 71.3, 69.8, 68.6, 60.2, 58.1, 56.1, 37.7, 29.8, 29.7, 29.1, 28.7, 27.7, 27.6, 23.5, 22.2, 22.1 (×2), 13.7, 10.3 (×2); ^{31}P NMR (CD_2Cl_2, 202 MHz) δ_P (ppm)= 12.18; FT-IR (ATR, σ/cm^{-1}): 3395, 2926, 1667, 1505, 1475, 1455, 1202, 1139, 1089. HRMS (−ESI, m/z): calcd. for $C_{43}H_{57}O_2N_8P_1{}^{32}S_1$ [M−H]$^-$: 792.3573; found, 792.3578. Anal. calcd. for $C_{43}H_{55}N_2O_8P_1S_1Na_2$ + $4H_2O$: C, 56.82.; H, 6.99; N, 3.08; P, 3.41 found: C, 56.21; H, 6.54; N, 2.71; P, 3.33.

Supporting Tables

Table S1. Maximum absorption wavelength (λ_{max}), maximum emission wavelength (λ_{em}), energy of the 0-0 transition (E_{00}) and Stokes shift ($\Delta\bar{\nu}$) of the different DPP dyes in DMF solution.

Dye	λ_{max} / nm	λ_{em} / nm	E_{00} / eV	$\Delta\bar{\nu}$ / cm^{-1}
**DPP1**	489	563	2.32	2688
**DPP2**	496	580	2.27	2920
**DPP3**	490	562	2.32	2615
**DPP4**	489	558	2.33	2529
**DPP5**	494	572	2.30	2760

Table S2. Dye loading experiments conducted on **DPP1** & **DPP4**.[a]

DPP	SED (pH)	[DPP] / µmol	Dye not attached to TiO_2 / % [b]	Dye attached to TiO_2 / % [c]
**DPP1**	AA (4.5)	0.05	19.4	80.6
		0.25	35.0	65.0
	TEOA (7.0)	0.05	0.0	100
		0.25	1.2	98.8
**DPP4**	AA (4.5)	0.05	1.2	98.8
		0.25	0.6	99.4
	TEOA (7.0)	0.05	0.0	100
		0.25	1.0	99.0

[a]3 mL samples were prepared by adding 50 or 250 µL of **DPP1 or DPP4** (1 mM in THF) to a stirred suspension containing 2.5 mg TiO_2 nanoparticles in an SED solution (2.95 or 2.75 mL). [b]The amount of dye remaining in the supernatant after centrifugation of TiO_2 (determined by comparing the UV-vis spectra of DPP before and after addition of TiO_2 and centrifugation). [c]The amount of dye attached to TiO_2 was determined indirectly: [Dye attached to TiO_2] = 100% – [Dye not attached to TiO_2].

Table S3. Control experiments for DSP with DPP│TiO_2│**CoP** and DPP│TiO_2│**NiP**.

Entry	System[a]	pH (buffer)	$TON_{catalyst}$ (after 2 h)	$n(H_2)$ / μmol (after 2 h)
1	**DPP1**│**NiP**, no TiO_2	4.5 (0.1 M AA)	1.8 ± 0.2	0.05 ± 0.01
2	**DPP4**│**NiP**, no TiO_2	4.5 (0.1 M AA)	–[b]	–[b]
3	**DPP1**│ZrO_2│**NiP**, ZrO_2 (2.5 mg) instead of TiO_2	4.5 (0.1 M AA)	–[b]	–[b]
4	**DPP4**│ZrO_2│**NiP**, ZrO_2 (2.5 mg) instead of TiO_2	4.5 (0.1 M AA)	0.5	0.01
5	**DPP1**│TiO_2, no **NiP**	4.5 (0.1 M AA)	–[b]	–[b]
6	**DPP1**│TiO_2, $NiBr_2 \cdot 3H_2O$ (0.025 μmol) instead of **NiP**	4.5 (0.1 M AA)	–[b]	–[b]
7	**DPP1**│TiO_2│**NiP**, no light	4.5 (0.1 M AA)	–[b]	–[b]
8	**DPP1**│TiO_2│**NiP**, no SED	6.8 (water)	–[b]	–[b]
9	**DPP1**│TiO_2│**NiP**, 0.1 M phosphate[c]	4.5 (0.1 M AA)	–[b]	–[b]
10	**DPP4**│TiO_2│**NiP**, 0.1 M phosphate[c]	4.5 (0.1 M AA)	–[b]	–[b]
11	**DPP1**│**CoP**, no TiO_2	7 (0.1 M TEOA)	–[b]	–[b]
12	**DPP4**│**CoP**, no TiO_2	7 (0.1 M TEOA)	–[b]	–[b]
13	**DPP1**│ZrO_2│**CoP**, ZrO_2 (2.5 mg) instead of TiO_2	7 (0.1 M TEOA)	–[b]	–[b]
14	**DPP1**│TiO_2, no **CoP**	7 (0.1 M TEOA)	–[b]	–[b]
15	**DPP1**│TiO_2, $CoBr_2 \cdot 3H_2O$ (0.05 μmol) instead of **CoP**	7 (0.1 M TEOA)	–[b]	–[b]
16	**DPP1**│TiO_2│**CoP**, no light	7 (0.1 M TEOA)	–[b]	–[b]
17	**DPP1**│TiO_2│**CoP**, no SED	6.8 (water)	–[b]	–[b]

[a]Experiments were performed at least in duplicate. Conditions: 2.5 mg TiO_2 or ZrO_2 as indicated, 0.025 μmol of **NiP** or 0.05 μmol of **CoP**, 0.05 μmol of dye, in 3 mL of SED solution as indicated. Samples were kept at 25 °C and irradiated with UV-filtered simulated solar light (100 mW cm^{-2}, AM 1.5G, λ > 420 nm) for at least 2 hours. [b]Below the limit of detection by gas chromatography. [c]KH_2PO_4 was added to the AA solution and the pH readjusted to pH 4.5.

Table S4. Optimisation of photocatalytic activity of DPP│TiO_2│**CoP** using **DPP1** and **DPP4** as light absorber and varying amounts of **CoP**.

Entry	System[a]	n (CoP) / µmol	n(H_2) / µmol (1 h)	TOF_{CoP} / h^{-1} (1 h)	TON_{CoP} (3 h)
18	**DPP1**│TiO_2│**CoP**	0.025	0.17 ± 0.02	6.8 ± 0.8	11.1 ± 1.1
19	**DPP1**│TiO_2│**CoP**	0.05	0.29 ± 0.03	5.8 ± 0.6	9.1 ± 0.6
20	**DPP1**│TiO_2│**CoP**	0.1	0.55 ± 0.06	5.5 ± 0.6	8.3 ± 0.8
21	**DPP1**│TiO_2│**CoP**	0.2	0.24 ± 0.03	1.2 ± 0.1	2.3 ± 0.2
22	**DPP4**│TiO_2│**CoP**	0.025	0.03 ± 0.01	1.1 ± 0.2	2.6 ± 0.2
23	**DPP4**│TiO_2│**CoP**	0.05	0.04 ± 0.01	0.8 ± 0.2	2.0 ± 0.2
24	**DPP4**│TiO_2│**CoP**	0.1	0.03 ± 0.01	0.3 ± 0.1	0.7 ± 0.2
25	**DPP4**│TiO_2│**CoP**	0.2	0.09 ± 0.02	0.5 ± 0.1	0.8 ± 0.1

[a]Experiments were performed at least in duplicate. Conditions: 2.5 mg TiO_2, varying amounts of **CoP**, 0.05 µmol of dye, 3 mL of TEOA SED solution (0.1 M, pH 7). Samples were kept at 25 °C and irradiated with UV-filtered solar light (100 mW cm^{-2}, AM 1.5G, λ > 420 nm).

Table S5. Comparison of photocatalytic activity of DPP│TiO_2│**CoP** under optimised conditions.

Entry	System[a]	n(H_2) / µmol (1 h)	TOF_{CoP} / h^{-1} (1 h)[b]	TON_{CoP} (3 h)[b]
26	**DPP1**│TiO_2│**CoP**	0.29 ± 0.03	5.8 ± 0.6	9.1 ± 0.6
27	**DPP2**│TiO_2│**CoP**	0.43 ± 0.04	8.8 ± 0.9	17.2 ± 1.7
28	**DPP3**│TiO_2│**CoP**	0.27 ± 0.03	5.4 ± 0.5	9.3 ± 0.9
29	**DPP4**│TiO_2│**CoP**	0.04 ± 0.01	0.8 ± 0.2	2.0 ± 0.2
30	**DPP5**│TiO_2│**CoP**	0.17 ± 0.02	3.4 ± 0.3	5.6 ± 0.6
31	**RuP**│TiO_2│**CoP**	1.42 ± 0.17	28.4 ± 3.4	48.4 ± 4.8

[a]Experiments were performed in triplicate. Conditions: 2.5 mg TiO_2, 0.05 µmol of **CoP**, 0.05 µmol of dye, 3 mL of TEOA SED solution (0.1 M, pH 7). Samples were kept at 25 °C and irradiated with UV-filtered solar light (100 mW cm^{-2}, AM 1.5G, λ > 420 nm). [b]$TOF_{dye} = 2 \cdot TOF_{CoP}$, $TON_{dye} = 2 \cdot TON_{CoP}$.

Table S6. Optimisation of photocatalytic activity of DPP│TiO_2│**NiP** using **DPP1** and **DPP4** as light absorber and varying amounts of **NiP**.

Entry	System[a]	n (NiP) / µmol	$n(H_2)$ / µmol (1 h)	TOF_{NiP} / h^{-1} (1 h)	TON_{NiP} (3 h)
32	**DPP1**│TiO_2│**NiP**	0.01	0.13 ± 0.01	13.3 ± 1.3	31.0 ± 3.1
33	**DPP1**│TiO_2│**NiP**	0.025	0.38 ± 0.04	14.7 ± 1.5	45.6 ± 4.6
34	**DPP1**│TiO_2│**NiP**	0.05	0.49 ± 0.11	9.8 ± 2.1	34.9 ± 6.5
35	**DPP1**│TiO_2│**NiP**	0.1	0.18 ± 0.02	1.8 ± 0.2	8.6 ± 0.9
36	**DPP4**│TiO_2│**NiP**	0.01	0.09 ± 0.01	9.3 ± 0.9	29.7 ± 2.9
37	**DPP4**│TiO_2│**NiP**	0.025	0.25 ± 0.03	10.0 ± 1.0	32.0 ± 3.2
38	**DPP4**│TiO_2│**NiP**	0.05	0.17 ± 0.03	3.4 ± 0.6	13.7 ± 3.1
39	**DPP4**│TiO_2│**NiP**	0.1	0.07 ± 0.01	0.7 ± 0.1	2.3 ± 0.5

[a]Experiments were performed at least in duplicate. Conditions: 2.5 mg TiO_2, varying amounts of **NiP**, 0.05 µmol of dye, in 3 mL of AA SED solution (0.1 M, pH 4.5). Samples were kept at 25 °C and irradiated with UV-filtered solar light (100 mW cm^{-2}, AM 1.5G, λ > 420 nm).

Table S7. Comparison of photocatalytic activity of DPP│TiO_2│**NiP** under optimised conditions.

Entry	System[a]	$n(H_2)$ / µmol (1 h)	TOF_{NiP} / h^{-1} (1 h)[b]	TON_{NiP} (21 h)[b]
40	**DPP1**│TiO_2│**NiP**	0.38 ± 0.04	14.7 ± 1.5	96.8 ± 9.7
41	**DPP2**│TiO_2│**NiP**	0.86 ± 0.09	34.6 ± 3.5	204.6 ± 20.5
42	**DPP3**│TiO_2│**NiP**	0.39 ± 0.04	15.5 ± 1.6	131.1 ± 13.1
43	**DPP4**│TiO_2│**NiP**	0.25 ± 0.03	10.0 ± 1.0	126.3 ± 12.6
44	**DPP5**│TiO_2│**NiP**	0.66 ± 0.07	26.4 ± 2.6	192.4 ± 19.2
45	**RuP**│TiO_2│**NiP**	1.35 ± 0.14	54.3 ± 5.4	233.6 ± 23.4

[a]Experiments were performed in triplicate. Conditions: 2.5 mg TiO_2, 0.025 µmol of **NiP**, 0.05 µmol of dye, 3 mL of AA SED solution (0.1 M, pH 4.5). Samples were kept at 25 °C and irradiated with UV-filtered solar light (100 mW cm^{-2}, AM 1.5G, λ > 420 nm). [b]$TOF_{dye} = 2 \cdot TOF_{NiP}$, $TON_{dye} = 2 \cdot TON_{NiP}$

Table S8. Results of EQE measurements for **DPP2**│TiO_2│**NiP** and **RuP**│TiO_2│**NiP** at different wavelengths.

Entry	System[a]	λ / nm	I / mW cm^{-2}	n(H_2) / µmol (2 h)	EQE / %
48	**DPP2**│TiO_2│**NiP**	400	3.05	0.029 ± 0.006	0.28 ± 0.06
49	**DPP2**│TiO_2│**NiP**	450	3.05	0.021 ± 0.002	0.17 ± 0.02
50	**DPP2**│TiO_2│**NiP**	475	3.13	0.028 ± 0.005	0.19 ± 0.03
51	**DPP2**│TiO_2│**NiP**	500	3.13	0.053 ± 0.005	0.41 ± 0.04
52	**DPP2**│TiO_2│**NiP**	550	3.05	0.022 ± 0.002	0.15 ± 0.02
53	**DPP2**│TiO_2│**NiP**	575	3.13	0.010 ± 0.002	0.07 ± 0.01
54	**RuP**│TiO_2│**NiP**	400	3.13	0.008 ± 0.001	0.08 ± 0.01
55	**RuP**│TiO_2│**NiP**	450	3.05	0.064 ± 0.006	0.53 ± 0.05
56	**RuP**│TiO_2│**NiP**	475	3.13	0.033 ± 0.003	0.23 ± 0.02
57	**RuP**│TiO_2│**NiP**	500	3.13	0.016 ± 0.002	0.12 ± 0.01
58	**RuP**│TiO_2│**NiP**	550	3.13	–[b]	–[b]

[a]Experiments were performed at least in duplicate at room temperature. Conditions: 2.5 mg TiO_2, 0.025 µmol **NiP**, 0.05 µmol of dye, 3 mL of AA SED solution (0.1 M, pH 4.5). [b] Below the limit of detection by gas chromatography.

Table S9. Comparison of photocatalytic activity of **DPP2**│TiO_2│H_2ase and **RuP**│TiO_2│H_2ase.

Entry	System[a]	TON_{H2ase} (1 h)	TON_{dye} (1 h)	n(H_2) / µmol (1 h)	TON_{H2ase} (21 h)	TON_{dye} (21 h)	n(H_2) / µmol (21 h)
59	**DPP2**│TiO_2│**H_2ase**	8650 ± 1100	17.3 ± 2.2	0.43 ± 0.06	87,600 ± 11,100	175 ± 22	4.38 ± 0.56
60	**RuP**│TiO_2│**H_2ase**	12,500 ± 1246	25.0 ± 3.5	0.62 ± 0.06	91,100 ± 22,300	182 ± 45	4.55 ± 1.11

[a]Experiments were performed in triplicate. Conditions: 2.5 mg TiO_2, 50 pmol [NiFeSe]-H_2ase, 0.05 µmol of dye, 3 mL of AA-MES solution (0.1 M each, pH 6). Samples were kept at 25 °C and irradiated with UV-filtered light (100 mW cm^{-2}, AM 1.5G, λ > 420 nm).

Table S10. Comparison of photocatalytic activity of **DPP2**│TiO_2│**Pt** and **RuP**│TiO_2│**Pt**.

Entry	System[a]	TON_{dye} (1 h)	n(H_2) / µmol (1 h)	TON_{dye} (24 h)	n(H_2) / µmol (24 h)
61	**DPP2**│TiO_2│**Pt** (AA)	337 ± 33.7	8.4 ± 0.8	2660 ± 265	66.4 ± 6.6
62	**RuP**│TiO_2│**Pt** (AA)	71.3 ± 7.1	1.8 ± 0.2	431 ± 95	10.8 ± 2.4

[a]Experiments were performed in triplicate. Conditions: 2.5 mg pre-platinised TiO_2, 0.05 µmol of dye, 3 mL of AA SED solution (0.1 M, pH 4.5) as indicated. Samples were kept at 25 °C and irradiated with UV-filtered solar light (100 mW cm^{-2}, AM 1.5G, λ > 420 nm).

Supporting Figures

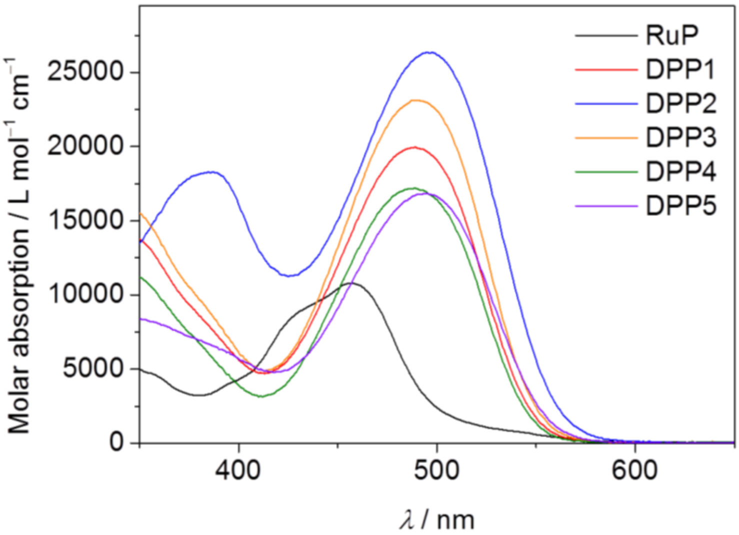

Figure S1. UV-Visible absorption spectra of DPP and **RuP** photosensitisers recorded in DMF.

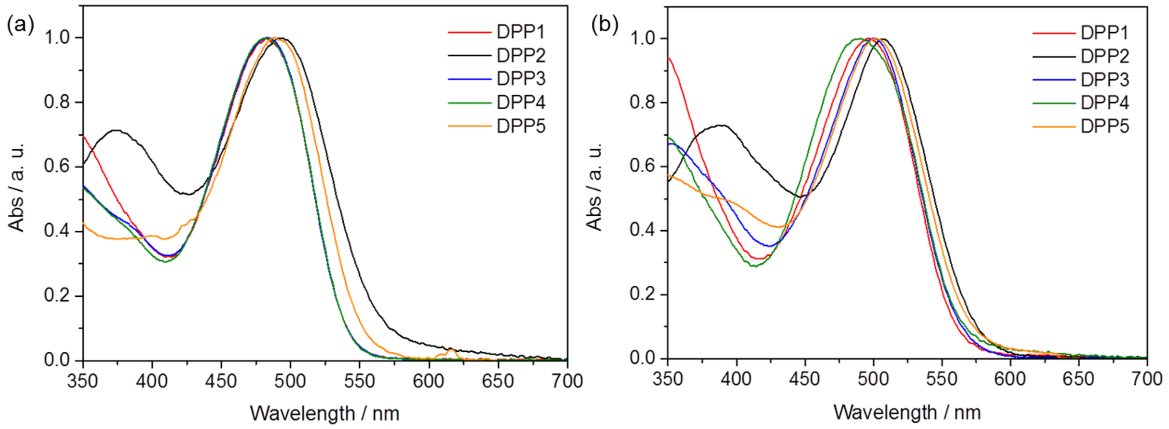

Figure S2. Normalised UV-Visible absorption spectra of diluted dyes **DPP1** to **DPP5** in (a) methanol and (b) toluene solution (cuvette path length = 1 cm).

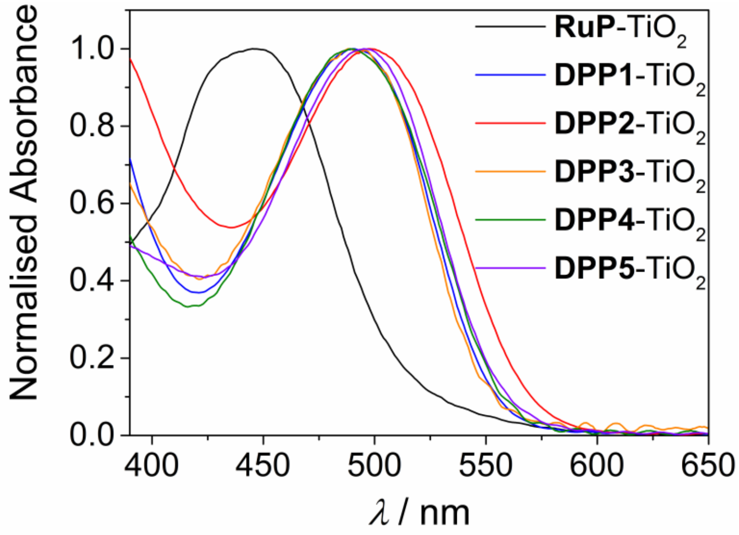

Figure S3. UV-Visible absorption spectra of DPP and **RuP** immobilised on mesoporous TiO_2 films.

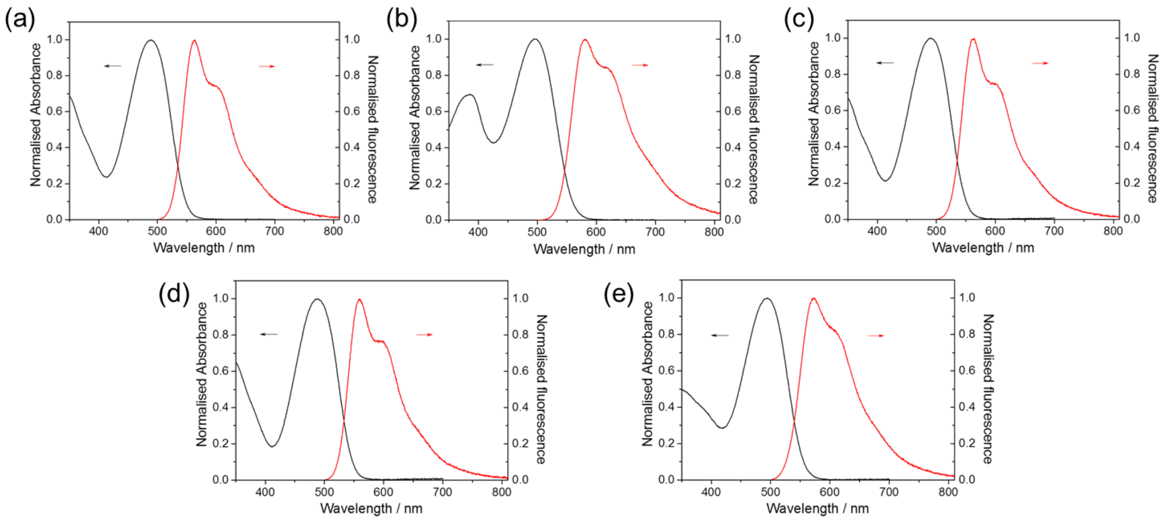

Figure S4. Normalised absorption and emission spectra (after excitation at 460 nm) of (a) **DPP1**, (b) **DPP2**, (c) **DPP3**, (d) **DPP4** and (e) **DPP5** in DMF (diluted solution, absorbance of ≈ 0.1, room temperature).

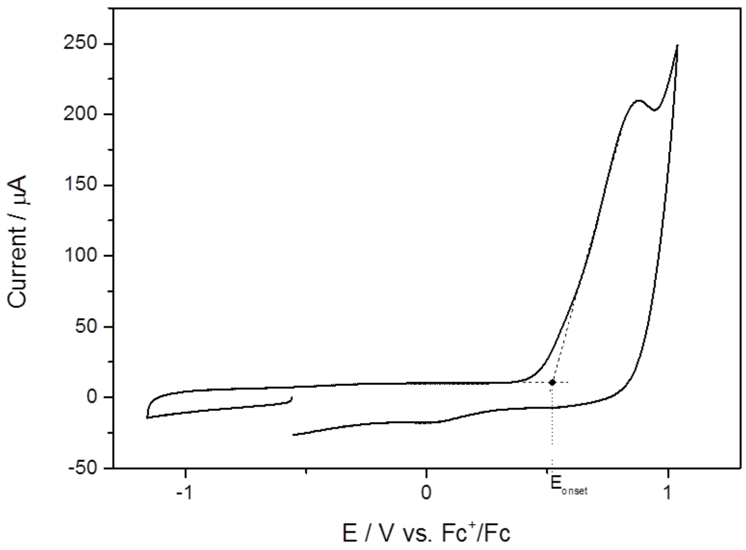

Figure S5. Representative cyclic voltammogram of **DPP1** chemisorbed on a mesoporous ITO electrode (0.1 M $TBABF_4$, scan rate 50 mV s^{-1}, room temperature).

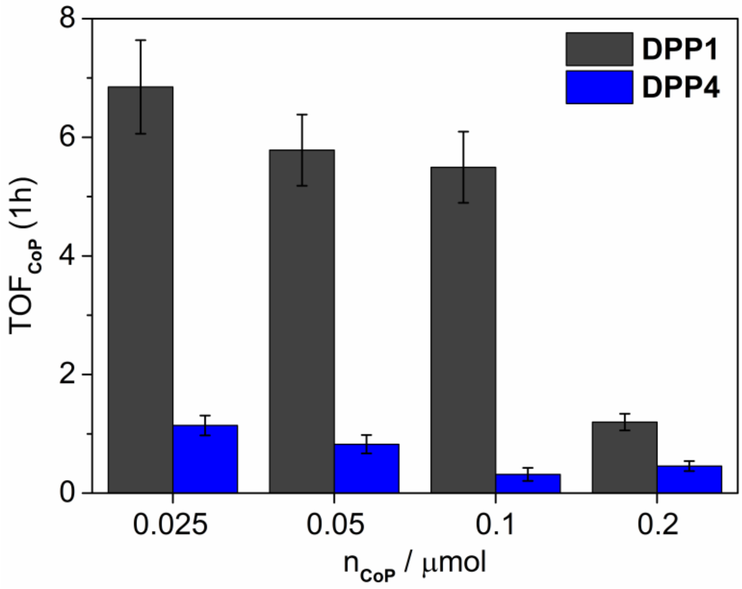

Figure S6. Optimisation of photocatalytic activity of DPP│TiO_2│**CoP** using **DPP1** and **DPP4** as light absorber and varying amount of **CoP**. The $TON_{\mathbf{CoP}}$ after one hour of UV-filtered simulated solar light irradiation (100 mW cm^{-2}, AM 1.5G, λ > 420 nm) is shown. Conditions: 2.5 mg TiO_2, varying amounts of **CoP**, 0.05 µmol of **DPP1** or **DPP4**, 3 mL TEOA solution (0.1 M, pH 7) at 25 °C (see Table S4).

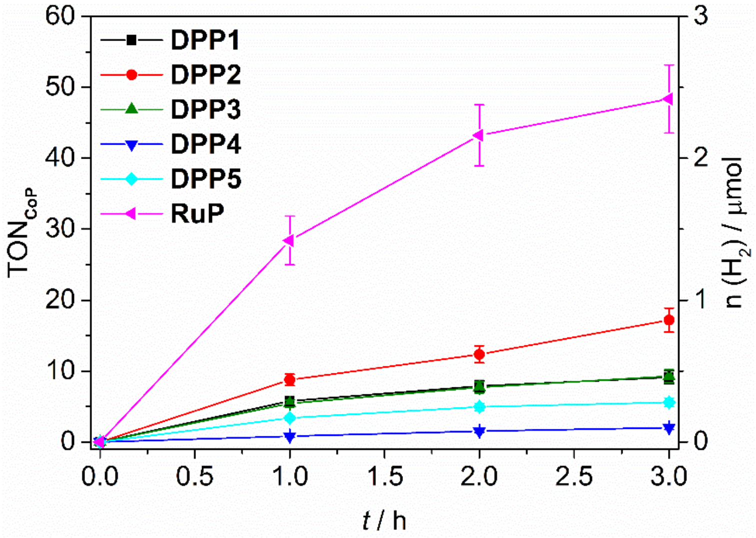

Figure S7. Comparison of photocatalytic activity of DPP|TiO_2|**CoP** and **RuP**|TiO_2|**CoP**. Conditions: 2.5 mg TiO_2, 0.05 µmol **CoP**, 0.05 µmol of DPP or **RuP**, 3 mL TEOA solution (0.1 M, pH 7), UV-filtered simulated solar light irradiation (100 mW cm^{-2}, AM 1.5G, λ > 420 nm) at 25 °C (see Table S5).

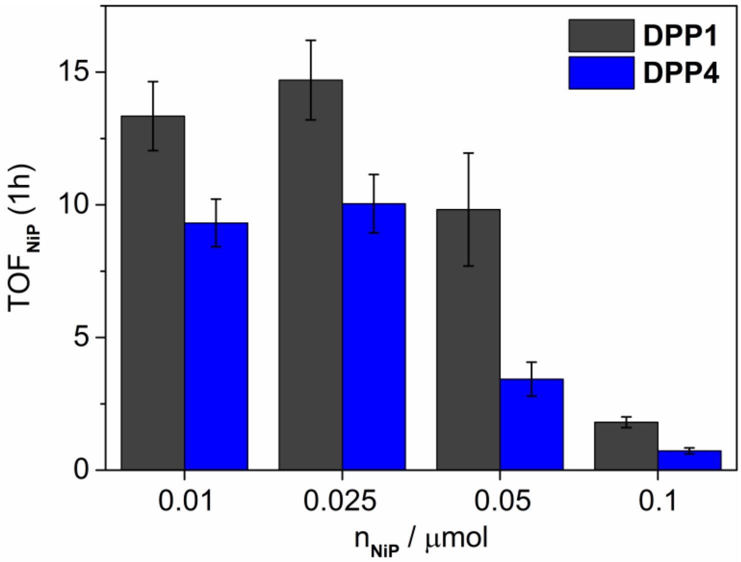

Figure S8. Optimisation of photocatalytic activity of DPP|TiO_2|**NiP** using **DPP1** and **DPP4** as light absorber and varying amount of **NiP**. The TON$_{NiP}$ after one hour of UV-filtered simulated solar light irradiation (100 mW cm^{-2}, AM 1.5G, λ > 420 nm) is shown. Conditions: 2.5 mg TiO_2, varying amounts of **NiP**, 0.05 µmol of **DPP1** or **DPP4**, 3 mL AA solution (0.1 M, pH 4.5) at 25 °C (see Table S6).

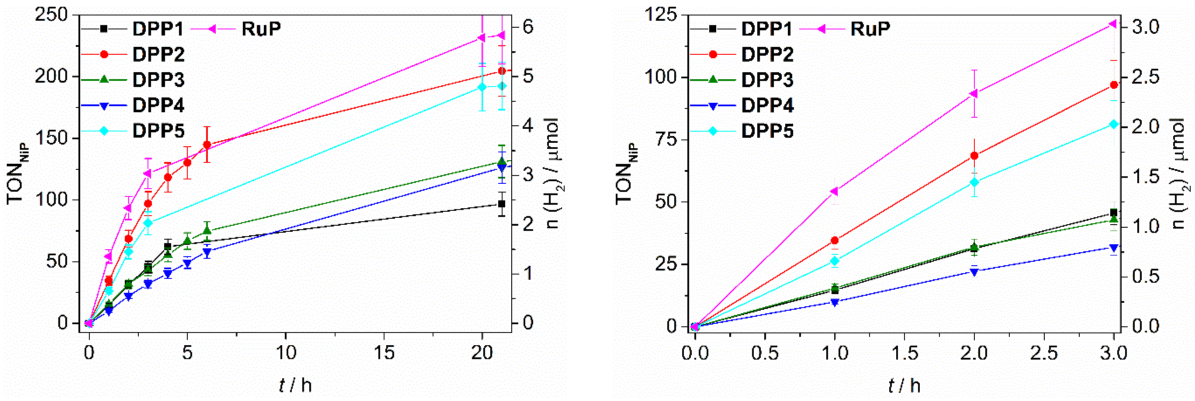

Figure S9. Comparison of photocatalytic activity of DPP|TiO_2|**NiP** and **RuP**|TiO_2|**NiP**. Conditions: 2.5 mg TiO_2, 0.025 µmol **NiP**, 0.05 µmol of DPP or **RuP**, 3 mL AA solution (0.1 M, pH 4.5), UV-filtered simulated solar light irradiation (100 mW cm^{-2}, AM 1.5G, λ > 420 nm) at 25 °C (see Table S7).

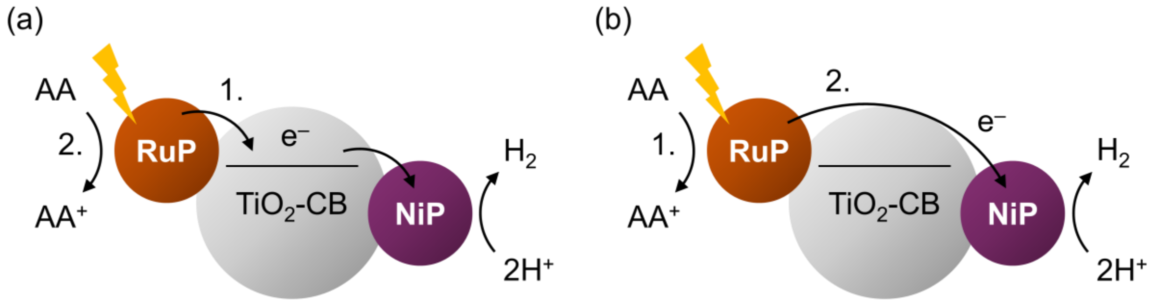

Figure S10. (a) Oxidative quenching pathway and (b) reductive quenching pathway in **RuP**|TiO_2|**NiP** as described previously.[2]

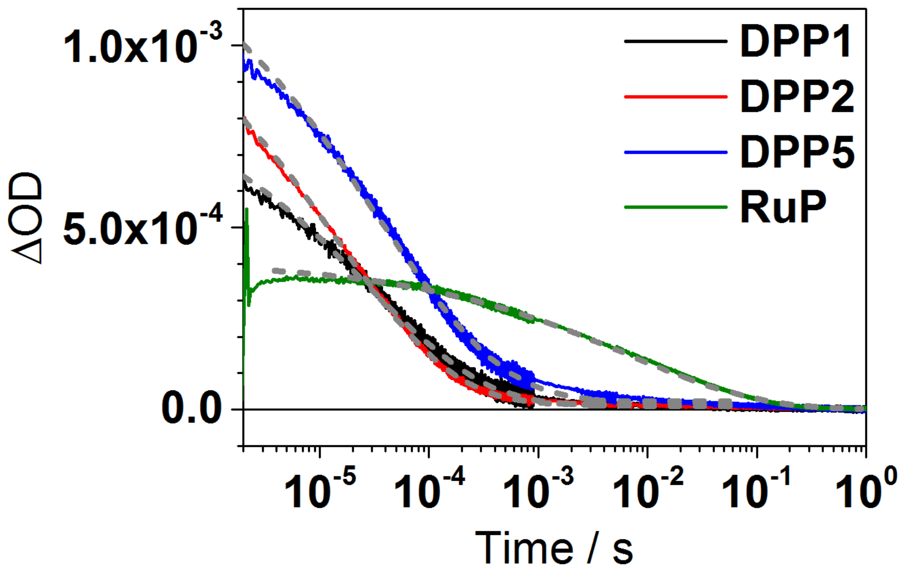

Figure S11. TAS decays monitored at 700 nm following photoexcitation of DPP-sensitised (λ_{ex} = 500 nm) or **RuP**-sensitised (λ_{ex} = 450 nm) TiO_2 thin films submerged in H_2O. Excitation fluences (ca. 200 µJ cm^{-2}) were adjusted to match the number of absorbed photons for all samples. Dashed grey lines represent stretched exponential fits to the data of the form $y = y_0 + A\exp\left(-\frac{t}{\tau_{kww}}\right)^{\beta}$. The resulting β stretch parameters were 0.35 – 0.40.

Supporting References

1. E. Bae and W. Choi, *J. Phys. Chem. B*, 2006, **110**, 14792-14799.
2. M. A. Gross, A. Reynal, J. R. Durrant and E. Reisner, *J. Am. Chem. Soc.*, 2014, **136**, 356-366.
3. F. Lakadamyali and E. Reisner, *Chem. Commun.*, 2011, **47**, 1695-1697.
4. I. Gillaizeau-Gauthier, F. Odobel, M. Alebbi, R. Argazzi, E. Costa, C. A. Bignozzi, P. Qu and G. J. Meyer, *Inorg. Chem.*, 2001, **40**, 6073-6079.
5. T. Bura, N. Leclerc, S. Fall, P. Lévêque, T. Heiser, P. Retailleau, S. Rihn, A. Mirloup and R. Ziessel, *J. Am. Chem. Soc.*, 2012, **134**, 17404-17407.
6. N. M. Muresan, J. Willkomm, D. Mersch, Y. Vaynzof and E. Reisner, *Angew. Chem. Int. Ed.*, 2012, **51**, 12749-12753.
7. J. Warnan, L. Favereau, Y. Pellegrin, E. Blart, D. Jacquemin and F. Odobel, *J. Photochem. Photobiol. A*, 2011, **226**, 9-15.
8. L. Duan, Y. Xu, M. Gorlov, L. Tong, S. Andersson and L. Sun, *Chem.--Eur. J.*, 2010, **16**, 4659-4668.

End of Electronic Supporting Information
